# Distinct roles for the domains of the mitochondrial aspartate/glutamate carrier citrin in organellar localization and substrate transport

**DOI:** 10.1016/j.molmet.2024.102047

**Published:** 2024-10-16

**Authors:** Sotiria Tavoulari, Denis Lacabanne, Gonçalo C. Pereira, Chancievan Thangaratnarajah, Martin S. King, Jiuya He, Suvagata R. Chowdhury, Lisa Tilokani, Shane M. Palmer, Julien Prudent, John E. Walker, Edmund R.S. Kunji

**Affiliations:** Medical Research Council Mitochondrial Biology Unit, University of Cambridge, Keith Peters Building, Cambridge Biomedical Campus, Hills Road, Cambridge, CB2 0XY United Kingdom

**Keywords:** Calcium regulation, Citrin deficiency, SLC25, Transport, Urea cycle disorders

## Abstract

**Objective:**

Citrin, the mitochondrial aspartate/glutamate carrier isoform 2 (AGC2), is structurally and mechanistically the most complex SLC25 family member, because it consists of three domains and forms a homo-dimer. Each protomer has an N-terminal calcium-binding domain with EF-hands, followed by a substrate-transporting carrier domain and a C-terminal domain with an amphipathic helix. The absence or dysfunction of citrin leads to citrin deficiency, a highly prevalent pan-ethnic mitochondrial disease. Here, we aim to understand the role of different citrin domains and how they contribute to pathogenic mechanisms in citrin deficiency.

**Methods:**

We have employed structural modeling and functional reconstitution of purified proteins in proteoliposomes to assess the transport activity and calcium regulation of wild-type citrin and pathogenic variants associated with citrin deficiency. We have also developed a double knockout of citrin and aralar (AGC1), the two paralogs of the mitochondrial aspartate/glutamate carrier, in HAP1 cells to perform mitochondrial imaging and to investigate mitochondrial localisation.

**Results:**

Using 33 pathogenic variants of citrin we clarify determinants of subcellular localization and transport mechanism. We identify crucial elements of the carrier domain that are required for transport, including those involved in substrate binding, network formation and dynamics. We show that the N-terminal domain is not involved in calcium regulation of transport, as previously thought, but when mutated causes a mitochondrial import defect.

**Conclusions:**

Our work introduces a new role for the N-terminal domain of citrin and demonstrates that dysfunction of the different domains contributes to distinct pathogenic mechanisms in citrin deficiency.

## Highlights

• We study 33 citrin missense mutations causing citrin deficiency.
• We identify elements crucial for transport within the carrier domain of citrin.
• The transport activity of citrin is not calcium-regulated, as previously thought.
• Pathogenic mutations in the N-terminal domain cause a mitochondrial import defect.
• Citrin domains contribute to distinct pathogenic mechanisms in citrin deficiency.

## Graphical abstract

**Figure:**
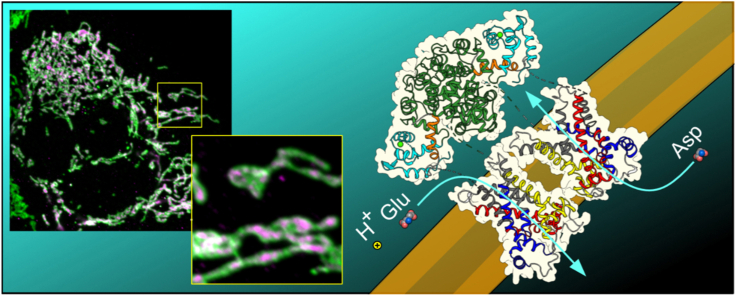

## 1 Introduction

The mitochondrial aspartate/glutamate carrier is located in the mitochondrial inner membrane and is responsible for the symport of glutamate and a proton into the mitochondrial matrix and the export of aspartate to the cytoplasm [1,2]. The carrier is an integral component of the malate/aspartate shuttle and provides important transport steps in several metabolic pathways, such as the urea cycle, gluconeogenesis, and energy metabolism [3]. There are two human paralogs of the carrier, aralar (AGC1 – encoded by the *SLC25A12* gene) [4] and citrin (AGC2 – encoded by the *SLC25A13* gene) [5], predominantly expressed in excitable and non-excitable tissues, respectively. Dysfunction of citrin causes citrin deficiency [6], one of the most prevalent mitochondrial diseases with a pathological gene occurrence of 1 in 42–65 in Japan and 1 in 28–45 in China, but patients are now found worldwide, as recently reviewed [3]. More than one hundred different pathogenic variants have been reported in the literature for citrin [3], which are more than those found in all other members of the SLC25 family combined [7].

Citrin deficiency is a complex disease with variable clinical manifestations in three age-related stages. In the first year of life, patients develop neonatal intrahepatic cholestasis caused by citrin deficiency (NICCD), characterized by jaundice, failure to thrive, hypoproteinemia, hypoglycemia, citrullinemia, other aminoacidemias, and fatty liver [[8], [9], [10]]. This period is followed by an adaptation stage when patients develop strong food preferences and present with milder clinical symptoms [10]. At this stage, many suffer from failure to thrive and dyslipidemia caused by citrin deficiency (FTTDCD) [11]. In adult life, a subset of patients develops adult-onset citrullinemia type II (CTLN2), the most severe disease form that can lead to death [6,8]. This phase may be characterized by frequent attacks of hyperammonemia, brain oedema, neuropsychiatric symptoms, liver steatosis and cancer [12].

In terms of structural and functional complexity, the mitochondrial aspartate/glutamate carriers are exceptional members of the SLC25 family. Unlike the others, they exist as homo-dimers [13] and each protomer has a three-domain architecture, composed of an N-terminal calcium-binding domain with eight EF-hands, followed by an SLC25 mitochondrial carrier domain responsible for substrate transport [1,14], and a C-terminal domain, composed of an amphipathic helix [13]. The roles of dimerization and the N- and C-terminal domains in the transport mechanism are not understood.

The structure of the full-length aspartate/glutamate carrier has not been elucidated, but the structural features of the carrier domain resemble those of the mitochondrial ADP/ATP carrier, available in different conformational states [[15], [16], [17]], and the uncoupling protein 1 [18,19]. The carriers consist of three homologous sequence repeats [20], forming a three-fold pseudo-symmetrical structure with a central translocation pathway for substrates [21]. They transport the substrate via an alternating access mechanism, interconverting between the matrix-open and cytoplasmic-open states [22,23]. The cytoplasmic-open to matrix-open state conversion is achieved by closing the cytoplasmic side of the carrier with three gate elements and by opening of the matrix side with three core elements in an alternating way. In the matrix-open to cytoplasmic-open state conversion, the same elements are involved but acting in reverse. The opening and closing are regulated by the disruption and formation of two salt bridge networks and braces: the matrix salt bridge network [16,17] and a glutamine brace [16] on the core elements and the cytoplasmic salt bridge network [15,22,24] and tyrosine braces [15] on the gate elements. These functional elements have not been studied before in the mitochondrial aspartate/glutamate carriers.

Previous studies have shown that the malate/aspartate shuttle activity in isolated mitochondria is calcium-regulated [14], suggesting that calcium binding could regulate the citrin or aralar transport activity directly. This notion was supported by sequence information indicating that the N-terminal domains of citrin and aralar contain eight EF-hands, usually associated with calcium-dependent regulatory mechanisms. To understand the role of the N-terminal domain better, atomic structures were solved in three different states [13]. In the first structure (PDB entry 4p5w), the N-terminal domain of citrin was fused with the C-terminal domain by a short linker loop, which replaced the carrier domain. The structure showed that the amphipathic helix of the C-terminal domain binds in a conserved cleft of the N-terminal domain. Strikingly, only EF-hand 2 binds calcium in a preformed binding site, whereas EF-hands 4–8 have evolved away from their canonical function to form a dimerization interface [13]. In the second and third structures, the N-terminal domain of aralar alone was solved in the absence or presence of calcium (PDB entries 4p60 and 4p5x, respectively). Comparison of the two aralar structures showed that in the absence of the C-terminal domain EF-hands 1 and 2 can rotate relative to the rest of the N-terminal domain in a calcium-dependent manner, where EF-hand 3 acts as a pivot point [13]. On this basis, EF-hands 1 and 2 were called the “mobile unit” whereas EF-hands 4–8 were called the “static unit”, also involved in dimerization. However, these calcium-dependent motions could only be confirmed in the absence of the C-terminal domain. Thus, the relevance of both the binding of calcium and the amphipathic helix to the transport mechanism of the entire complex had not been clarified.

Here, we use more than thirty pathogenic missense mutations found in citrin deficiency for structural modelling, transport assays, and mitochondrial imaging to obtain mechanistic insights into the function of citrin. First, we identify residues responsible for substrate binding, network formation and conformational dynamics in the carrier domain. Second, we show that the N-terminal domain does not regulate transport activity via calcium binding, as previously thought. Instead, we show for the first time that pathogenic mutations in the N-terminal domain of citrin cause a mitochondrial localization defect that is unrelated to calcium binding in EF-hand 2.

## 2 Materials and methods

### 2.1 Structural model of citrin

**Figure 1:**
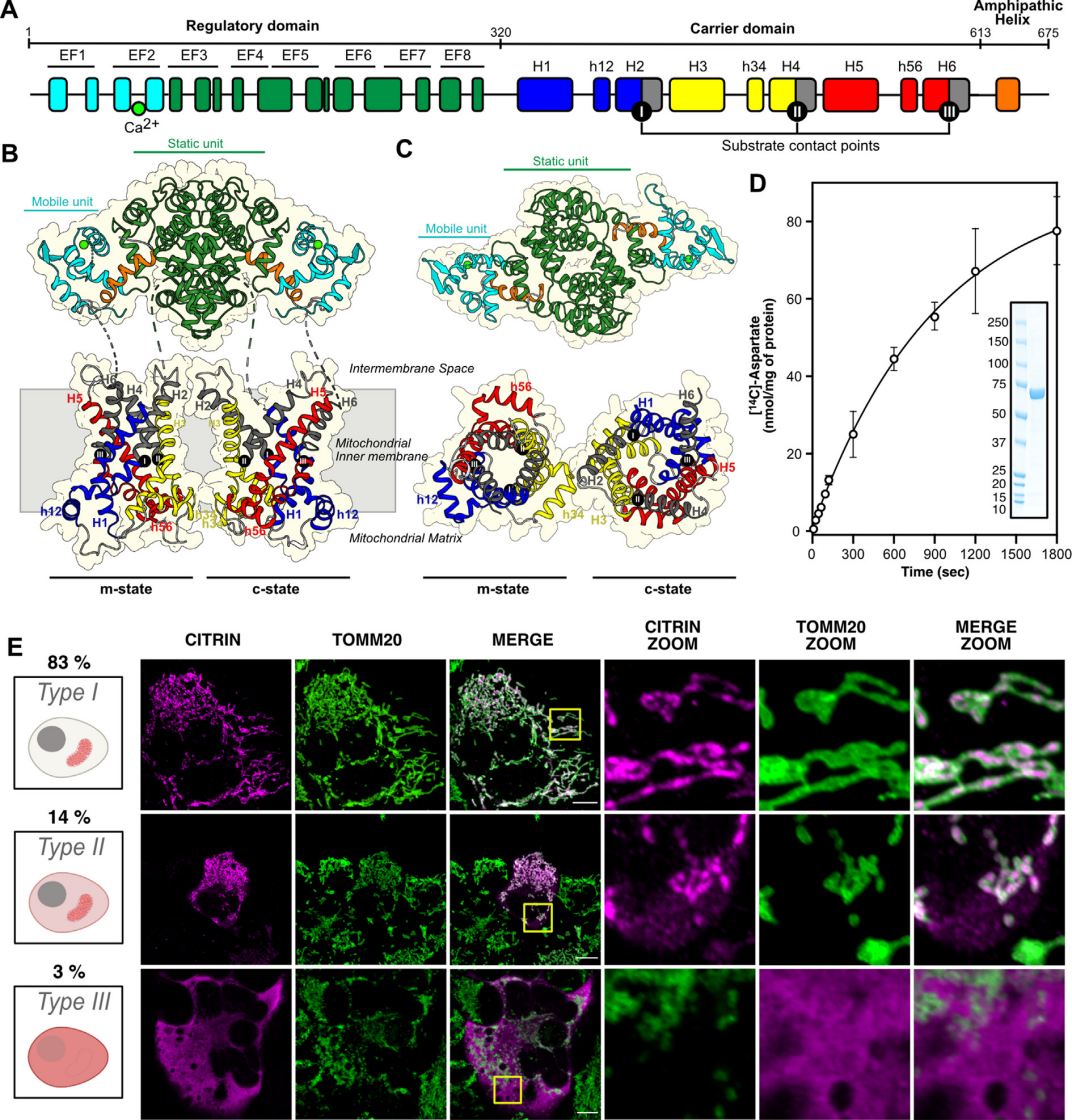
**Experimental systems to study function and mitochondrial localization of citrin pathogenic variants.** (**A**) Domain structure of citrin. In the N-terminal domain the calcium-bound mobile unit and static unit are shown in cyan and green, respectively. In the carrier domain the three core elements are shown in blue, yellow and red and the three gate elements in gray. The C-terminal domain is shown in orange. The residue boundaries between the domains are indicated. (**B–C**) Lateral and cytoplasmic view of the citrin structural model in the same color scheme as in (**A**). (**D**) Transport activity measured with purified citrin reconstituted into proteoliposomes. SDS-PAGE and Coomassie staining of full-length wild type is shown in the inset. (**E**) Representative confocal images of HAP1 citrin and aralar DKO expressing wild-type citrin transiently. Upper row: Type I cells with citrin localization exclusively in mitochondria. Middle row: Type II cells with mixed localization of citrin in mitochondria and cytoplasm. Bottom row: Type III cells with primarily cytoplasmic localization of citrin. Citrin immunostaining (first column), TOMM20 immunostaining of mitochondria (second column), and merge (third column). Respective zoomed areas are shown in the fourth, fifth and sixth columns. Scale bars are at 10 μm.

To evaluate the structural context of the pathogenic mutations, a structural model of the citrin carrier domain (Q9UJS0) (Δ1-328 and Δ607-675) in the cytoplasmic-open state was generated by SWISS-MODEL, based on the yeast ADP/ATP carrier (PDB entry 4c9g) [25]. A structural model of the citrin carrier domain in the matrix-open state was generated by SWISS-MODEL (Q9UJS0), based on the ADP/ATP carrier of *Thermothelomyces thermophila* (PDB entry 6gci). For mutations in the regulatory domain, the experimentally determined atomic structure was used (PDB entry 4p5w). The structural regions linking the regulatory domain to the carrier domain and the carrier domain to the C-terminal domain are unknown. As such, the relative positions of the domains are also not defined (Figure 1B). Each pathogenic mutation was introduced with the mutagenesis wizard function in PYMOL [26] by considering the most frequent backbone-restrained conformer not causing a steric clash. The wild-type and mutant structures were visualized with PYMOL [26].

### 2.2 Molecular biology

The coding sequence for human citrin isoform NP_055066.1 (Uniprot: Q9UJS0) was synthesized (GenScript) and subcloned into the pcDNA3 vector for expression in mammalian cells. For yeast expression [27], a codon-optimized cDNA of human citrin was synthesized (GenScript) and subcloned into vectors pYES3/CT or pYES2/CT (Invitrogen), where carrier domain or N-terminal domain mutations were introduced, respectively. The codon-optimized AGC2 also carries an N-terminal eight-histidine tag followed by the sequence coding for a TEV protease cleavage site (ENLYFQG) for full-length AGC2 or a factor Xa cleavage site (DAAIEGRTSED) for the carrier domain alone. Mutagenesis, in the mammalian and yeast expression vectors, was performed by overlap-extension PCR [28] with KOD HotStart polymerase (Novagen).

### 2.3 Yeast expression and mitochondrial isolation

The expression plasmids were transformed into *Saccharomyces cerevisiae* strain W303-1B (MATα *leu2-3, 112 trp1-1 can1-100 ura3-1 ade2-1 his3-11,15*), by the LiAc/SS carrier DNA/PEG method [29]. Successful transformants were selected on synthetic-complete tryptophan-dropout (pYES3/CT) or uracil-dropout (pYES2/CT) medium (Formedium) supplemented with 2% (w/v) glucose. Large-scale expression was performed as described previously [13].

### 2.4 Protein purifications

Full-length citrin and its carrier domain alone (Δ1-319 and Δ616-675) were purified as described before with minor modifications [13]. Immediately prior to purification, 0.5 g of mitochondria were thawed and suspended in buffer containing 20 mM HEPES, pH 8.0, 150 mM NaCl, 10% (*v/v*) glycerol, one Complete EDTA-free protease inhibitor cocktail tablet (Roche) and 1% (*w/v*) lauryl maltose neopentyl glycol (LMNG, Anatrace). Mitochondria were solubilized for 1.5 h at 4 °C with gentle agitation and the insoluble material was removed by ultracentrifugation at 205,000×*g* for 45 min. The supernatant was incubated for 2 h with nickel Sepharose High Performance beads (Cytiva), in the presence of 20 mM imidazole, and then transferred into an empty column (Bio-Rad). The resin was washed initially with 25 column volumes of buffer A (20 mM HEPES, pH 8.0, 300 mM NaCl, 50 mM imidazole, 10% glycerol, 0.1% (*w/v*) LMNG, 0.1 mg/mL tetraoleoyl cardiolipin (TOCL, Avanti Polar Lipids)), followed by 20 column volumes of buffer B (20 mM HEPES, pH 8.0, 150 mM NaCl, 10% glycerol, 0.1% (*w/v*) LMNG, 0.1 mg/mL TOCL). The protein was retrieved from the column by on-column digestion for 12 h at 10 °C with 125 μg of MBP-TEV protease (full-length citrin) or at 4 °C with 15 μg of factor Xa (carrier domain), followed by removal of the resin with empty Proteus Midi spin columns (Generon) at 200×*g* for 5 min. Subsequently, TEV protease was removed by incubation of the purified protein with 250 μL amylose resin (New England Biolabs). 7K cut-off Zeba spin desalting columns (ThermoFisher) were used to remove imidazole and exchange to buffer C (20 mM MES pH 6.4, 150 mM NaCl, 0.1% (*w/v*) LMNG, 0.1 mg/mL TOCL). Removal of calcium for calcium-free conditions was achieved by incubating the purified protein with 50 mM EGTA and 50 mM EDTA for 30 min followed by desalting using Zeba 7K columns. The columns were previously equilibrated with Buffer B containing 50 mM EDTA and EGTA then washed with 20 mM HEPES, pH 7.4, 150 mM NaCl, 5 mM EGTA, 0.1% (*w/v*) LMNG, 0.1 mg/mL TOCL. Protein concentrations were determined using a Nanodrop spectrophotometer (ThermoFisher). Purified proteins are shown in Figure S1. The protein bands were resolved by SDS-PAGE, excised from the gel and identified by peptide mass fingerprinting using liquid chromatography MS/MS.

### 2.5 Protein reconstitution in proteoliposomes

Egg l-α-phosphatidylcholine and TOCL (Avanti Polar Lipids) were mixed in a 30:1 (w/w) ratio, dried under a stream of nitrogen and hydrated in 20 mM HEPES, pH 7.4 at 15 mg/mL. Unlabeled aspartate was added at a final concentration of 5 mM for internalization. Lipids were solubilized with 1.8% (v/v) pentaethylene glycol monodecyl ether (Sigma) and freshly purified protein was added at a lipid:protein ratio of 250:1 (w/w). Liposomes were formed by the stepwise removal of pentaethylene glycol monodecyl ether by five additions of 60 mg Bio-Beads SM-2 (Bio-Rad) at 20-minute intervals with gentle mixing at 4 °C, followed by addition of 480 mg Bio-Beads overnight. Proteoliposomes were first separated from the Bio-Beads and collected by passage through empty spin columns (Bio-Rad).

To prepare proteoliposomes in calcium-free conditions, *E. coli* polar lipid extract (Avanti Polar Lipids), egg l-α-phosphatidylcholine (Avanti Polar Lipids) and tetraoleoyl cardiolipin (Avanti Polar Lipids) were mixed in a 15:5:1 (w/w) ratio and were dried and processed as above with the exception that they were solubilized with 2.6% pentaethylene glycol monodecyl ether (Sigma). The lipid:protein ratio was maintained at 250:1. Additionally, the following modifications were applied; the protein was treated with EGTA/EDTA, as described in section 2.4 and all buffers were prepared with commercially available calcium-free water (Supelco) and calcium-free chemicals. Additional decontamination was achieved through incubation with Chelex 100 resin (3 g/L). Furthermore, 5 mM EGTA was added in the reconstitution mixture to ensure that traces of calcium still present were chelated. To ensure the integrity of the lipids, 3 mM ultra-pure MgCl_2_ was added during reconstitution to replace calcium.

### 2.6 Substrate transport in proteoliposomes

The aspartate/glutamate carrier is a strict exchanger and has no efflux activity [1]. The external substrate was removed from the formed proteoliposomes by passing them through a PD10 column. They were then diluted six times in assay buffer (20 mM HEPES, pH 7.4, 1 mM CaCl_2_) and 100 μL were loaded onto a MultiScreenHTS + HA 96-well filter plate (pore size 0.45-μm, mixed cellulose ester, Millipore (#MSHAN4B)) on a Hamilton MicroLab Star robot (Hamilton Robotics Ltd). Uptake of radiolabeled [^14^C]-aspartic acid (American Radiolabeled Chemicals) was initiated by 100 μL assay buffer containing 2 μM [^14^C]-aspartic acid. The uptake was stopped by filtration and washing three times with 200 μL ice-cold assay buffer. Levels of radioactivity were measured in 200 μL MicroScint-20 (Perkin Elmer) in a TopCount scintillation counter (Perkin Elmer). The signal of each variant was normalized against citrin wild type based on SDS-PAGE analysis of the proteins after reconstitution in proteoliposomes. Uptake curves were fitted with one phase exponential and initial rates were determined from the linear part (GraphPad Software).

For transport in calcium-free conditions, all buffers were passed through Chelex resin. The total amount of calcium remaining was measured by total calcium analysis using an 8900 Triple Quadrupole Inductively Coupled Plasma Mass Spectrometer (ICP-MS; Agilent Technologies, USA Trace Element Analysis Facility, part of the Department of Veterinary Medicine Research Facilities at the University of Cambridge). The instrument was set up to scan in MS/MS mode with the reaction cell gas in He (Helium) gas mode. Calcium was measured at the following mass transitions, 43 -> 43 and 44 -> 44, but only the former was used for calculation of Ca concentration. Calibration standards were prepared with a Ca ICP standard solution (1,000 ppm Ca; Fluka Ltd). All samples and blanks (media alone) were diluted 1 to 5 in ultra-high purity water. A reference material (MEE NIST REF 1643) was analysed alongside the diluted samples and calibration standards and gave 101.3% recovery. The Chelex-treated buffers were then treated with 5 mM EGTA, and supplemented with magnesium, which does not become chelated by EGTA as efficiently as calcium and can sustain the integrity of the liposomes. The free-calcium concentration was estimated using Maxchelator (https://somapp.ucdmc.ucdavis.edu/pharmacology/bers/maxchelator/). The following parameters were used to estimate the final concentration of free calcium present in the samples: Temperature: 15 °C, pH: 7.4, and ionic strength: 10 mM (the final concentrations of MgCl2 and EGTA were 1.5 mM and 5 mM, respectively, leading to an ionic strength below 10 mM).

For normalization of the transport signal, calibration curves were established by analyzing increasing concentrations of purified wild-type citrin protein or the purified carrier domain, as appropriate, on SDS-PAGE. These curves were used to quantify the amount of protein reconstituted in proteoliposomes and normalize the uptake.

### 2.7 Protein expression and purification in *L. lactis* and analysis of oligomeric state by size exclusion chromatography coupled to multiangle laser light scattering

A fusion of the N- and C-terminal domains of citrin (residues 2–319 and 613–675, respectively), containing an N-terminal octa-histidine tag and TEV protease cleavage site (ENLYFQG) [13] was used to introduce mutation G176V via overlap-extension PCR. The fusion protein was expressed and purified from *Lactococcus lactis* NZ9000, as described [13]. Size exclusion chromatography coupled to multiangle laser light scattering (SEC-MALLS) was performed with a Superdex 200 10/300 GL column (GE Healthcare) coupled in-line with a light scattering detector (Dawn HELEOSII, Wyatt Technologies) and a refractive index detector (Optilab T-rREX, Wyatt Technologies), as described [13]. The protein was injected onto a column equilibrated with 20 mM HEPES, pH 7.4, 150 mM NaCl and 5 mM CaCl_2_. All data were recorded and analyzed with ASTRA 6.03 (Wyatt Technologies). The molecular weights of the N- and C-terminal domain fusion of citrin and the G176V mutant were determined by using the standard two-detector method.

### 2.8 Thermostability analyses

Protein folding was assessed by two methods, thermal denaturation in the presence of 7-diethylamino-3-(4-maleimidophenyl)-4-methylcoumarin (CPM) on a rotary quantitative PCR (qPCR) instrument (Qiagen Rotor-Gene Q 2plex HRM) or nano-differential scanning fluorimetry (nano-DSF) using the NanoTemper Prometheus NT.48 (NanoTemper). In the CPM method, cysteine residues, buried within the protein structure, become solvent exposed during denaturation in a temperature ramp and form fluorescent adducts with CPM. CPM working solution was prepared by diluting a 5 mg/mL CPM stock made in dimethyl sulfoxide 50-fold into assay buffer (20 mM MES pH 6.4, 150 mM NaCl, 0.1% (*w/v*) LMNG, 0.1 mg/mL TOCL) and incubated for 10 min at room temperature. About 3 μg of purified citrin were diluted into 45 μL assay buffer, incubated for 10 min on ice and then 5 μL of the CPM working solution were added. Samples were incubated on ice and in the dark for a further 10 min and then subjected to a temperature ramp of 5.6 °C/min. The fluorescence increase was monitored with the High Resolution Melt (HRM) channel (excitation at 440–480 nm, emission at 505–515 nm). Unfolding profiles were analyzed with the Rotor-Gene Q software 2.3. For nano-DSF, protein samples in buffer containing 20 mM MES pH 6.4, 150 mM NaCl, 0.1% (*w/v*) LMNG, 0.1 mg/mL TOCL, were loaded into capillary tubes. A temperature ramp of 5 °C/min was applied and the intrinsic fluorescence was measured in the NanoTemper Prometheus NT.48 (NanoTemper).

### 2.9 Generation of aralar and citrin double knock-out HAP1 cells

HAP1 cells where both citrin and aralar had been knocked out were generated as described previously [30]. Briefly, one pair of gRNAs used to target exon I of each gene by CRISPR/Cas9 gene editing, were cloned into plasmid pSpCas9(BB)-2A-GFP, and each 0.5 μg DNA of two pairs of the resultant plasmids was transfected into HAP1 cells simultaneously by lipofectamine 3000 (ThermoFisher Scientific) and sorted a single cell into 96-well plates via GFP fluorescence. The clones were screened by immunoblotting with antibodies against citrin (ab96303) and aralar (ab200201) (Abcam). The exon I regions of the edited clones were amplified by PCR, gel purified and determined by Sanger sequencing. The gRNA sequences used were 5′-CGAGCACAGCATGGCGGTCA-3′ and 5′-AGGTCTTAGATAGCTTGTCC-3′ for the *SLC25A12* gene. For *SLC25A13* they were 5′-GCCATGATTCGCCCCGGTTG-3’ (-, reverse complement) and 5′-CGGGCCCGCGGTTACCTTGG-3’ (−)*.* To PCR amplify each disrupted exon I region for Sanger sequencing, the primers forward 5′-CCCAAAGCCACACCCACTAA-3′ and reverse 5′-CCGTACAAGCCCCTTCAACT-3′ were used for *SLC25A12*; forward 5′-GCTTGCACACGGCCAAGTTA-3′ and reverse 5′-CAAAGTTTCCGCTGCGAGG-3′ were used for *SLC25A13.*

### 2.10 Cell culture and transfection of HAP1 cells

HAP1 cells were obtained from the American Type Culture Collection (ATCC) and cultured in Iscove's Modified Dulbeccco's Medium + Glutamax, supplemented with 10% fetal bovine serum (FBS) (all from GIBCO) at 37 °C with 5% CO_2_. The cells were routinely tested for mycoplasma using the Lookout Mycoplasma PCR detection kit (Sigma). Prior to seeding, 13 mm round glass coverslips were transferred to a standard 24-well plate and coated with 30 μL/cm^2^ poly-lysine at 37 °C for 15 min. After rinsing and drying of the coverslips, cells were seeded at a density of 20 × 10^5^ per coverslip and allowed to attach and recover for 24 h. Cells were transfected with 250 ng of plasmid DNA at 1:4 ratio with Turbofectin 8.0 (Origene) following the manufacturer's instructions, and fixed 24 h post transfection.

### 2.11 Immunostaining

Immunostaining was performed as described [31]. Briefly, the cells were fixed in 5% paraformaldehyde in PBS at 37 °C for 15 min, then washed three times with PBS, followed by quenching with 50 mM ammonium chloride in PBS. After three washes in PBS, cells were permeabilized in 0.1% Triton X-100 in PBS for 10 min, followed by three washes in PBS and then blocked with 10% FBS in PBS for 30 min, followed by incubation with primary antibodies in 5% FBS in PBS, for 2 h at room temperature. After 3 washes with 5% FBS in PBS, cells were incubated with the secondary antibodies (1:1,000) for 1 h at room temperature, followed by three washes in PBS. The coverslips were then mounted onto slides using Dako fluorescence mounting medium (Dako). Citrin was detected using the N-terminal primary antibody HPA018997 (Human Protein Atlas) (1:250) and an anti-rabbit secondary antibody conjugated with Alexa Fluor 488. TOMM20 was detected with the primary 11802-1-AP (ThermoFisher Scientific) (1:1,000) and a secondary conjugated with Alexa Fluor 647.

### 2.12 Confocal imaging

Stained cells were imaged using either a 60X or 100X objective lens (NA1.4) on a Nikon Eclipse TiE inverted microscope with appropriate lasers using an Andor Dragonfly 500 confocal spinning disk system, equipped with a Zyla 4.2 PLUS sCMOS camera coupled with Fusion software (Andor). Seven stacks of 0.2 μm each were acquired using the 100X objective. Images acquired in the same conditions of laser intensity and exposure time from the same experiment were then compiled by “max projection” and analyzed using FIJI. For signal quantification, individual cells were manually segmented, and then specific parameters were extracted from each channel, i.e. citrin and TOMM20 staining, using FIJI. Data are shown as the ratio of citrin standard deviation over the mean grey value normalized to the integrated TOMM20 intensity, adjusted to 1 × 10^10^. Ten fields of view per condition were analyzed in each of the three to four independent experiments, using Fiji software [32]. The number of cells analyzed are shown in Table S1.

### 2.13 Super-resolution imaging

For super-resolution Structured Illumination Microscopy (SIM), images from fixed samples were captured using a Nikon SIM microscope equipped with an SR Apo TIRF 100 × 1.49 N A. oil objective and a DU897 Ixon camera (Andor). Eleven z-stacks, each 0.2 μm thick, were obtained using appropriate laser lines. The raw images were computationally reconstructed with the 3D-SIM stack reconstruction algorithm provided by NIS-Elements software (Nikon). Line-scans were carried out in ImageJ by drawing a line across the width of a mitochondrial segment demarcated by the TOMM20 signal. The “Plot profile” function in ImageJ was used to generate the relative fluorescence intensity for all channels. These values were normalized to the maximum intensity value (arbitrary units) of each channel and plotted on the x-axis against the distance of the bisecting line (1 μm), represented on the y-axis. The data were then visualized and plotted using GraphPad Prism.

### 2.14 Statistical analysis

Hierarchical clustering of cell type distribution was performed on the Morpheus web application (Broad Institute, USA), assuming one minus Pearson correlation and average linkage method. The threshold was then set to show a maximum of five clusters.

For immunostaining signal quantification, a two-way ANOVA without interaction was used to assess main variant effect followed by a two-stage linear step-up procedure of Benjamini, Krieger and Yekutieli to adjust for multiple comparisons. Statistical analyzes were performed using Graph Pad Prism version 9.5.1 (GraphPad Software). Differences were considered significant at 5% level (Table S2).

## 3 Results

### 3.1 *In silico*, *in vitro* and cellular models to study citrin function and mitochondrial localization

We developed a structural model of citrin, based on the known structures of the ADP/ATP carrier [15,16] and the previously determined structure of the N-terminal and C-terminal domain fusion of citrin [13] (Figure 1A,B,C). The structural model was then used to predict essential functional and structural elements, based on prior knowledge from other SLC25 mitochondrial carriers [7,15,22,23,[33], [34], [35]]. We also established two experimental approaches, one to study the transport activity of the citrin pathogenic variants with purified proteins reconstituted into liposomes (Figure 1D, Figure S1), and the other to study their expression and mitochondrial localization in human cells (Figure 1E). For the latter, we constructed a double citrin and aralar knock-out (DKO) HAP1 cell line (Figure S2), and in this background we expressed either wild-type citrin or one of 33 different pathogenic variants. For the mitochondrial localization studies, we observed three different cell types within a population based on citrin staining and localization: Type I; cells with exclusive mitochondrial staining, Type II; cells with mitochondrial and non-mitochondrial staining and Type III; cells with primarily non-mitochondrial staining of citrin (Figure 1E). For wild-type citrin, most cells were Type I, as analyzed by the co-localization of citrin with the mitochondrial outer membrane marker, TOMM20. Only a small fraction of cells corresponded to Type II and III, 14.0 ± 5.0% and 2.8 ± 4.3%, respectively. The same analysis was performed on all variants retrieved from the literature [3]**.** Strikingly, we observed marked differences in the mitochondrial localization for several of them, as discussed further in the following sections. For approximately two-thirds of variants, D39T, F96S, G333D, R355G, R355Q, G386V, G393S, K405N, V411M, G436E, E450G, R455L, K453R, V474M, C489R, D493G, R585H, G531D, A541D, T546R, R553Q, Q592P, E601K, there was a mitochondrial localization in most transfected cells (Type I). However, for the other eleven variants, there was a large proportion of cells with non-mitochondrial staining. Except for five, A25E, D39T, G176V, R588P and Q592P, the overall expression level of most of the missense variants was similar to that of wild-type citrin in HAP1 cells (Table S2).

### 3.2 Critical functional elements in the carrier domain of citrin

**Figure 2:**
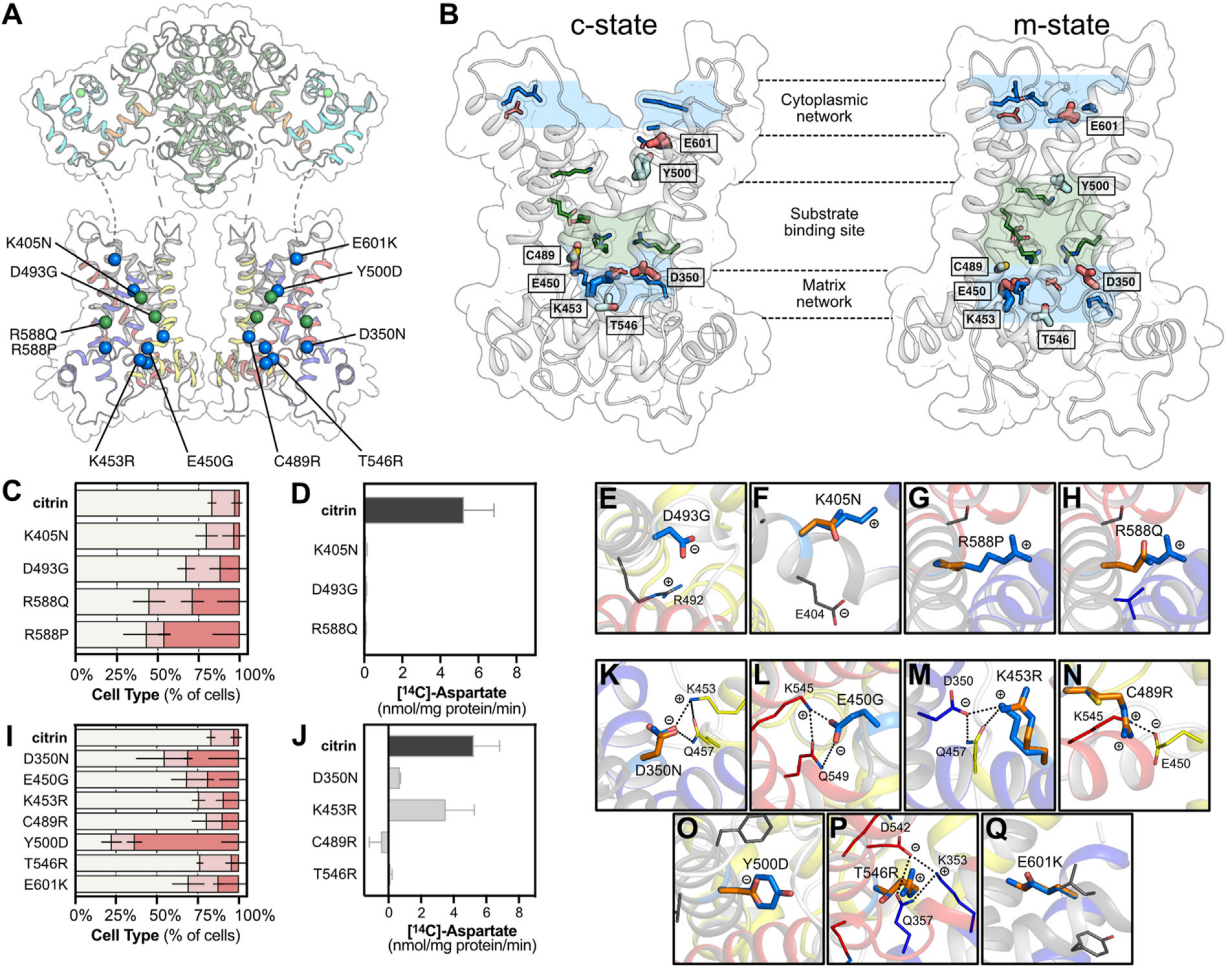
**Critical functional elements in the carrier domain of citrin.** (**A**) Pathogenic mutations affecting substrate binding and salt bridge networks are shown in green and blue spheres, respectively. (**B**) Carrier domain of citrin in c-state (left) and m-state (right) with networks and binding site residues shown in sticks. Thicker sticks indicate the mutated residues involved directly or indirectly in the cytoplasmic or matrix network. (**C, I**) Mitochondrial and cytosolic distribution of citrin and disease variants, classified into three types, Type I: mitochondrial, Type II: dual, Type III: cytosolic (n = 7 for citrin wild type, n = 3 for variants). Relative frequencies of each cell type (%) are indicated with different colors (Type I cells in beige, Type II in salmon, Type III in red). The number of cells analyzed is shown in Table S2. (**D, J**) Time course of aspartate homo-exchange. Initial rates of transport for wild type were compared with those of pathogenic variants (n = 12 for citrin wild type, n = 2–3 for variants). (**E-H, K-Q**) Pathogenic mutations, shown in their structural contexts. The wild-type residues are shown as marine sticks and the mutations in orange. n represents independent biological repeats. The error bars represent the standard deviation in transport assays (panels D, J) and the standard error of the mean in distribution assays (panels C, I).

First, we focused on identifying residues of the central cavity that contribute to substrate binding. As with other SLC25 family members, the substrate binding site is predicted to have three contact points on helices H2, H4 and H6 [7,[35], [36], [37]] (Figure 1A,B and C). We studied four citrin pathogenic mutations located on those helices, having side chains facing the cavity and potentially coordinating the substrate, specifically K405N, D493G, R588Q and R588P (Figure 2A,B). Although K405N and D493G had no considerable effect on mitochondrial localization in human cells (Figure 2C), they completely abolished substrate transport (Figure 2D). Both mutations at position R588 (R588Q and R588P) increased the proportion of Type II and III cells (Figure 2C and Figure S3). More importantly, R588Q, despite its conservative nature, abolished transport completely (Figure 2D). These results are consistent with the idea that these residues have an essential role in substrate coordination. Indeed, K405 along with E404 on H2 correspond to contact point I (Figure 1A,B and Figure 2F), where K405 was predicted to bind the carboxylate side chain of aspartate and glutamate [36,37] and E404 was proposed to be involved in proton translocation [38]. D493 and R492 on H4 (Figure 2E) are part of contact point II and could be binding the amino group of the substrates, whereas R588 on H6 in contact point III (Figure 2G,H) could also be interacting with the carboxyl group of the substrate [36,37].

We then focused on residues that are involved in the matrix or cytoplasmic salt bridge networks, which are key mechanistic features of all SLC25 mitochondrial carriers [7,15,[22], [23], [24],^33^,^34^]. The formation and disruption of these networks regulate access to the central substrate binding site by closing and opening the carrier to the matrix side or cytoplasmic side in an alternating way. Five pathogenic variants are likely to affect the matrix network (D350N, E450G, K453R, C489R and T546R) directly or indirectly, whereas E601K could be part of the cytoplasmic network and Y500D could be in its proximity (Figure 2A,B). Most of these mutations had little or no effect on the proportion of Type II and III cells (Figure 2I and Figure S4), but they had variable effects on activity (Figure 2J), likely explained by the nature of the substitution and their position. The participation of the relevant positions in the formation of the two networks is shown in Figure S5. D350N in the matrix network (Figure 2K) abolishes the transport activity almost entirely by directly affecting the interactions within the network (Figure S5), whereas K453R (Figure 2M) retains a moderate transport rate, because it is a conservative replacement. Although C489 is not part of the matrix network, C489R (Figure 2N) could disrupt it indirectly via steric hindrance or bonding with E450 (Figure 2N). T546 is also not part of the matrix network, but T546R introduces a charged arginine that could disrupt key interactions within the network, eliminating activity (Figure 2P).

In the cytoplasmic network, E601 on H6 (Figure 2Q) forms a salt bridge interaction with K508 on H4 during the formation of the cytoplasmic network in the matrix-open state (Figure S5). With the replacement of the negative charge with a positive one in the E601K mutation, the formation of this cytoplasmic network could be prevented. Below the cytoplasmic network are aromatic and hydrophobic residues that act as an insulation layer when the carrier is in the matrix state and the cytoplasmic network is formed [15]. Y500D (Figure 2O) introduces a small and negatively charged residue into this layer, which had a strong effect on increasing the number of Type III cells (Figure 2I and Figure S4), the only one of all carrier domain variants to have such a strong effect. This effect could occur because of a severe property mismatch of the two residues or because a mitochondrial targeting signal, which is currently unknown, is affected. Both Y500D and E601K could not be assessed for transport activity, as it was not possible to produce them in sufficient yields, again indicating issues with biogenesis.

### 3.3 Critical dynamic and structural elements in the carrier domain of citrin

**Figure 3:**
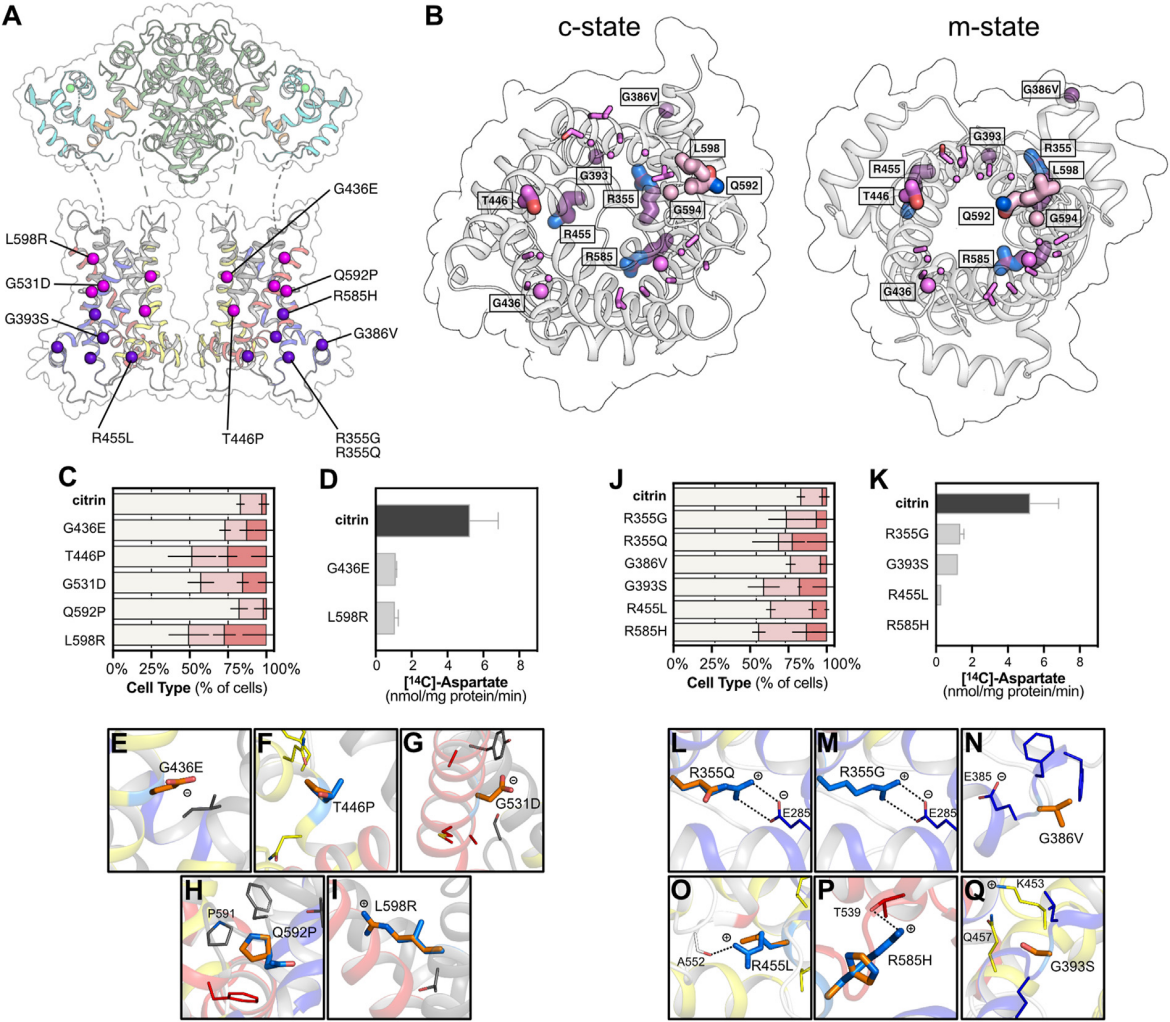
**Residues important for structural stability and dynamic changes of the carrier domain.** (**A**) Pathogenic mutations affecting dynamic changes or structural features are shown in pink and purple spheres, respectively. (**B**) Carrier domain of citrin in c-state (left) and m-state (right) with residues highlighted as sticks. (**C, J**) Distribution of mitochondrial and cytosolic citrin and disease variants, classified into three types, Type I: mitochondrial, Type II: dual, Type III: cytosolic (n = 7 for citrin wild type, n = 3 for variants). Relative frequencies of each cell type (%) are indicated with different colors as in Figure 2. The number of cells analyzed are shown in Table S2. (**D, K**) Time course of aspartate homo-exchange. Initial rates of transport for wild-type and pathogenic variants (n = 12 for citrin wild type, n = 2–3 for variants). (**E-I, L-Q**) Citrin pathogenic mutations, shown in their structural contexts. The wild-type residues are shown in marine and mutations in orange. n represents biological repeats. The error bars represent the standard deviation in transport assays (panels D, K) and the standard error of the mean in distribution assays (panels C, J).

Interhelical interfaces in members of SLC25 generally contain small residues important for the transition between the cytoplasmic-open and matrix-open states [15,23] (Figure 3A,B), but when mutated they could prevent the conformational changes [7]. We have identified some of these residues in citrin, which are mutated in citrin pathogenic variants. In G436E, the percentage of Type II and III cells was similar to that of wild-type citrin. G531D and L598R increased the numbers of Type II and III cells and reduced transport activity to less than 20% of wild type (Figure 3C,D). Both G436E and G531D introduce a negatively charged amino acid in place of glycine (Figure 3E,G). L598R introduces a large and positively charged residue into a small and hydrophobic interhelical space, which could jam the carrier dynamics (Figure 3I). Other pathogenic mutations affecting protein dynamics are T446P and Q592P (Figure 3F,H), which had a moderate or no effect on mitochondrial localization, respectively (Figure 3C and Figure S6). Mutations to proline interfere with the hydrogen bonding arrangement of helices, but their functional characterization was not possible.

Other structurally important residues in the carrier domain could be residing in loops, loop-to-helix transitions or in sites for tightly bound cardiolipins, as observed for other family members [15,16]. We studied six citrin pathogenic variants where mutations are found in these positions (Figure 3A,B). Most of them increased the percentage of Type II and III cells only modestly (Figure 3J and Figure S7). However, the transport functions of all variants were strongly affected (Figure 3K). R455L and R585H had no transport activity, whereas R355G and G393S had less than 25% residual activity. However, these proteins were obtained with very low yields, probably due to structural instabilities, which could have affected the functional analyses. These strong effects on activity are supported by the structural model, as R355 could be forming an important ionic interaction with E285, linking transmembrane H1 with matrix h12, which would be abolished in the R355Q and R355G mutations (Figure 3L,M). The G386V mutation might interfere with the matrix helix h12 to loop transition, destabilizing the domain structure (Figure 3N). R455 forms a key interaction with carbonyl backbone of A552, which is abolished in R455L (Figure 3O). R585 forms an important ionic interaction with T539 (Figure 3P) and probably with D542 as well, which could be abolished by the R585H mutation, affecting the structure and matrix network. G393S might be preventing the binding of the phosphate moiety of cardiolipin disturbing the loop to helix transition (Figure 3Q) or this substitution might inadvertently interact with Κ453 and Q457, which are part of the matrix salt bridge network and glutamine brace, respectively, interfering with their function in the mechanism.

### 3.4 The bound calcium does not regulate the transport mechanism or affect citrin mitochondrial localization

It had been previously proposed that citrin and aralar transport activities are stimulated by calcium [1,14,39], although the molecular mechanism of this effect has not been established. Crystal structures of the aralar N-terminal domain alone show a conformational transition between the calcium-free and the state where a single calcium is bound in EF-hand 2, but it is not clear whether this change is relevant to the regulation of the transport activity, as the carrier and C-terminal domains were absent [13].

**Figure 4:**
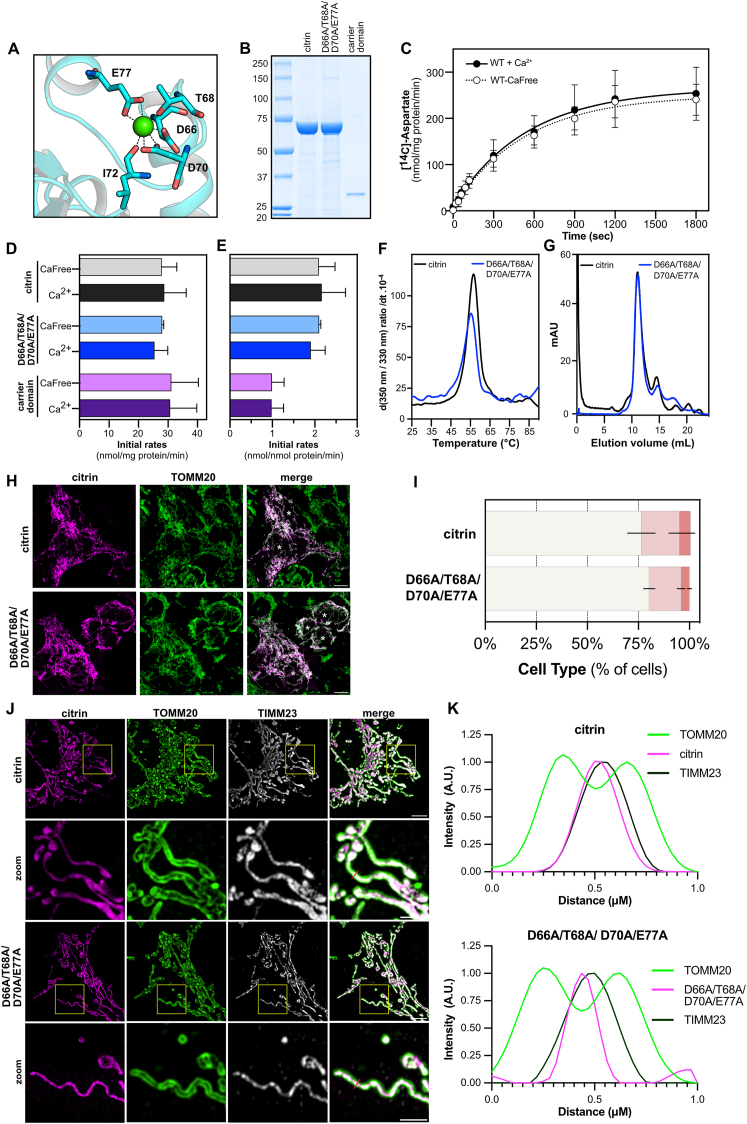
**Calcium does not regulate citrin activity or affect dimerization.** (**A**) Calcium-binding site of citrin in the presence of calcium, taken from the structure (PDB entry 4p5w). Calcium is shown as a green sphere and the interacting residues as sticks. (**B**) SDS-PAGE of wild-type citrin, the calcium-binding site mutant D66A/T68A/D70A/E77A and the carrier domain alone, purified with nickel affinity chromatography. (**C**) Time course of aspartate homo-exchange for wild-type citrin in the presence or absence of calcium (representative experiment repeated in three biological repeats). (**D**) Initial rates of transport (n = 3) in 1 mM calcium or calcium-free conditions presented in nmols of substrate per mg of protein. (**E**) Initial rates of transport (n = 3) in 1 mM calcium or calcium-free conditions in nmols of substrate per nmol of protein. (**F**) Unfolding curves of wild-type citrin and the calcium-binding site mutant via nano-differential scanning fluorometry (representative experiment repeated in three biological repeats). (**G**) Size exclusion chromatography for the wild-type citrin and the calcium-binding site mutant. In panels C–E, error bars represent the standard deviation. (**H**) Representative confocal images of HAP1 citrin and aralar DKO expressing transiently wild-citrin or D66A/T68A/D70A/E77A. For comparison, the signal intensity has been adjusted to that observed in cells expressing wild-type citrin. Left column: citrin immunostaining, Middle column: TOMM20 immunostaining of mitochondria, Right column: merge. Type I cells are indicated by an asterisk (∗). Scale bars: 10 μm. (**I**) Distribution of mitochondrial and cytosolic citrin, classified into three types (n = 3). Relative frequencies of each cell type (%) are indicated with different colors as in Figure 2. (**J**) Representative super-resolution structured illumination microscopy (SR-SIM) images of HAP1 citrin and aralar DKO expressing transiently wild-type citrin or D66A/T68A/D70A/E77A. Left column: citrin immunostaining, Middle column: TOMM20 immunostaining of mitochondria, Right column: translocase of inner mitochondrial membrane 23 (TIMM23). Far right panel is merged image. Zoomed insets can be seen below the corresponding images. Scale bar is at 5 μm or 2.5 μm for Zoom panels. Bisecting line (red): 1 μm. (**K**) Line-scan analysis of the relative fluorescence intensity of TOMM20, citrin or D66A/T68A/D70A/E77A and TIMM23 staining at the region marked by the bisecting line demonstrating an overlap of the citrin signal with the mitochondrial inner membrane marker TIMM23.

Here, we evaluated the effect of calcium on the transport activity of human citrin directly with purified protein, reconstituted into liposomes. We also expressed and purified the carrier domain alone and a quadruple alanine mutant in the single calcium-binding site in EF-hand 2 (D66A/T68A/D70A/E77A) (Figure 4A,B), which should completely abolish ion coordination. To achieve calcium-free conditions without compromising the integrity of the proteoliposomes (Figure S8), we chelated calcium with EGTA and replaced it with magnesium. Citrin was fully functional in calcium concentrations in the low picomolar range with 5 mM EGTA (Table S3) and did not respond to calcium, even at high concentrations of 1 mM (Figure 4C,D, E). To exclude the possibility that this effect is due to calcium being replaced by magnesium, we also tested the D66A/T68A/D70A/E77A mutant and found that it is also fully active (Figure 4D,E). These experiments showed that the transport activity is not regulated by calcium binding. Moreover, the carrier domain alone was also active (Figure 4D,E), suggesting that the presence of the N-terminal domain is not required for substrate transport.

We then considered whether calcium binding maybe be important for structural integrity or for dimerization. However, the D66A/T68A/D70A/E77A variant was purified with yields similar to that of wild-type citrin, and was folded, as established by thermostability assays (Figure 4F). Additionally, mutation of the calcium site did not affect the dimeric state, as the quadruple mutant and wild-type citrin both eluted as dimers in size exclusion chromatography (Figure 4G).

Next, we evaluated whether the abolishment of calcium binding affects the protein mitochondrial localization or its import into the inner mitochondrial membrane. First, we tested the quadruple alanine mutant at the calcium site for its ability to localize to mitochondria using confocal microscopy. No difference in mitochondrial localization was observed between wild-type citrin and D66A/T68A/D70A/E77A (Figure 4H,I). Additionally, we used super resolution microscopy to show that D66A/T68A/D70A/E77A, as well as wild-type citrin, are imported in the inner mitochondrial membrane (**Figure J, K**).

### 3.5 Pathogenic mutations of the N-terminal domain affect mitochondrial localization

**Figure 5:**
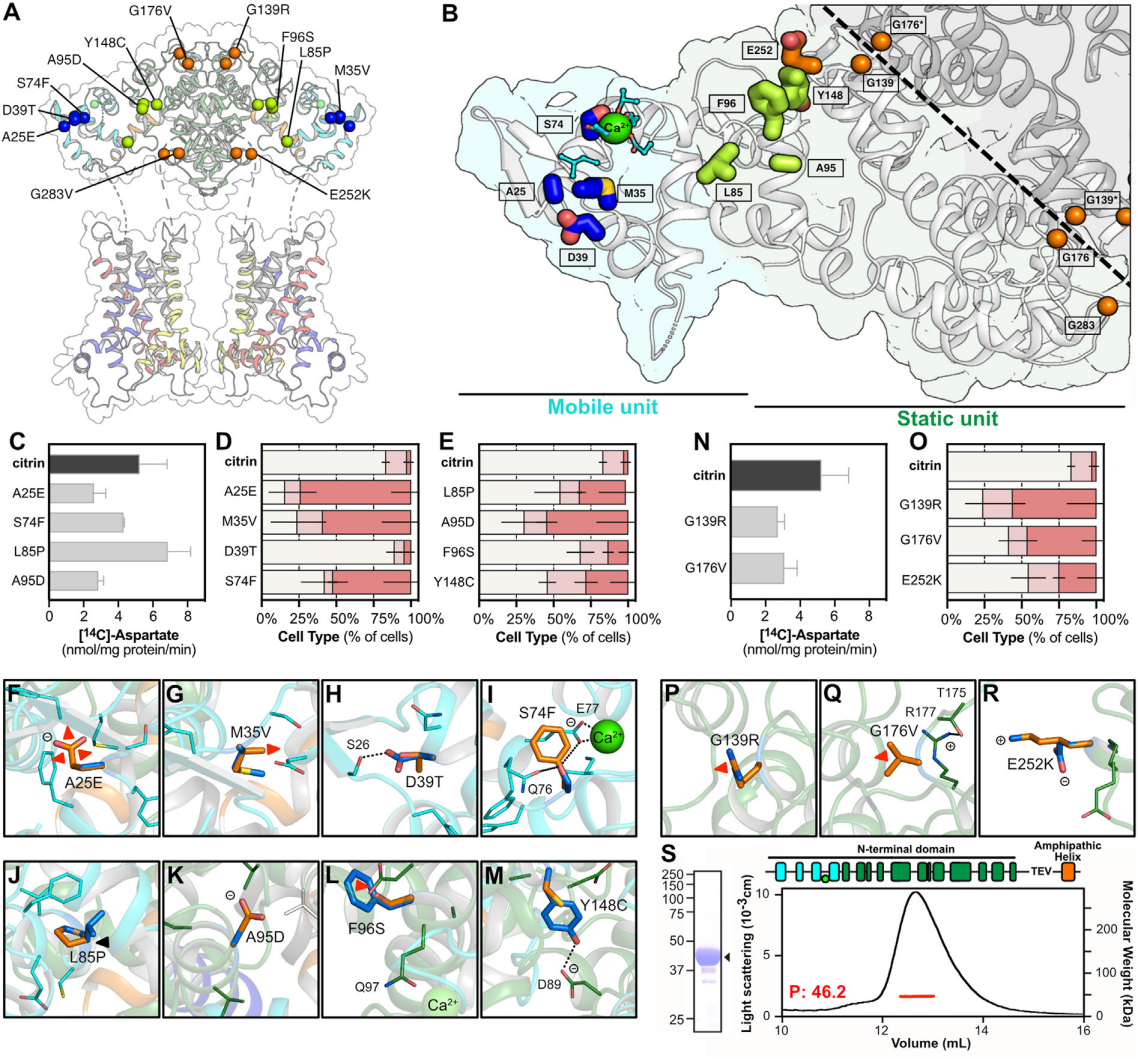
**Pathogenic mutations of the N-terminal domain affect mitochondrial localization.** (**A**) Residues in the N-terminal domain mutated in pathogenic variants. The ones located in the dimer interface, hinge region and mobile unit are shown in orange, green and dark blue spheres, respectively. (**B**) Close up view of the N-terminal domain with residues in stick representation, colored as in **A**. (**D, E, O**) Mitochondrial and cytosolic distribution of citrin and disease variants, classified into three types Type I: mitochondrial, Type II: dual, Type III: cytosolic (n = 7 for citrin wildtype, n = 3 for variants). Relative frequencies of each cell type (%) are indicated with different colors, as in Figure 2. The number of cells analyzed are shown in Table S2. (**C, N**) Time course of aspartate homo-exchange. Initial rates of transport compared for wild-type and the pathogenic variants indicated (n = 12 for citrin wild type, n = 2–3 for variants). (**S**) Oligomeric state analysis by SEC-MALLS for the N-terminal/C-terminal fusion containing G176V. (**F–M, P–R**) Citrin pathogenic mutations, shown in their structural context. Residues are shown in marine and mutations in orange. n represents biological repeats. The error bars represent the standard deviation in transport assays (panels C, N) and the standard error of the mean in distribution assays (panels D, E, O).

Our observations that the N-terminal domain is not absolutely required for citrin activity suggest that there could be yet another, unidentified explanation for the twelve pathogenic mutations in this domain, and thus we investigated them further. These pathogenic mutations affect different parts of the N-terminal domain (Figure 5A,B). S74F and L85P can be found in the interface between EF-hand 1 and EF-hand 2 in the “mobile” unit and “hinge” region of the N-terminus, respectively (Figure 5A,B). Both variants had initial transport rates that were similar to those of wild-type citrin (Figure 5C), showing that the transport function of the carrier domain was essentially unaffected. Variants A25E and A95D, in the mobile unit and hinge region, respectively, had initial transport rates of approximately 50% of the wild-type protein (Figure 5C), but they were produced in low yields and did not behave properly in reconstitutions. The most interesting and unexpected observation was that most of these variants had disrupted localization patterns, with Type III cells dominating in A25E, M35V, S74F and A95D, while L85P and Y148C had a more modest increase in the proportion of Type II and III cells (Figure 5D,E and Figs. S9 and S10). Hence, most of these mutants failed to localize properly to mitochondria.

Since the structure of the N-terminal domain is available we can assess the effect of each mutation on the local structure. A25E and M35V could lead to amino acid side chain clashes and to structural destabilization of EF-hand 1 (Figure 5F,G). D39T could be disrupting a hydrogen bond with S26, possibly leading to a local rearrangement of the loop of EF-hand 1 (Figure 5H). S74F could affect calcium binding indirectly as this residue interacts with neighboring Q76 and E77, which in turn form a key interaction with the calcium ion bound to EF-hand 2 (Figure 5I). L85P causes a truncation of one of the two helices of EF-hand 2 (Figure 5J) and A95D introduces a larger and negatively charged side chain into a hydrophobic pocket, both potentially causing structural instability (Figure 5K). F96S introduces a smaller polar residue, which could displace the helix and therefore the position of Q97 interacting directly with the calcium ion (Figure 5L). Finally, Y148C could be abolishing a key interaction between the hydroxyl group of Y148 with D89, possibly also destabilizing the structure (Figure 5M). Therefore, based on consideration of the functional and localization data, these structural defects primarily affect mitochondrial localization, but also the transport activity of citrin moderately in some cases. Subsequently, we tested whether mutations of the N-terminal domain, which have different localisation profiles, impair protein folding, resulting in defective protein import as a secondary effect. Using thermostability analyses, we showed that mutants A25E, S74F, L85P are clearly folded, albeit with slightly lower melting temperatures (Figure S12). Their purification samples also showed the presence of Hsp60 (Figure S12). A95D had a more complex unfolding profile indicative of folding issues, which could account for the low yield of this variant. Interestingly, the carrier domain, expressed and purified separately, was fully folded and even more thermostable than the carrier domain of the wild-type citrin (Figure S12).

### 3.6 Pathogenic mutations in the dimerization interface also affect citrin localization

Mutations G139R, G176V, and E252K are in the dimerization interface of the homodimer (Figure 5A,B) and the role of dimerization in citrin function is currently unknown. Interestingly, although both G139R and G176V proteins were produced with very low yields, they had initial transport rates approximately half that of the wild-type carrier (Figure 5N). G139R, and to a lesser extend G176V, showed a big increase in Type II and III cells, whereas E252K was affected modestly (Figure 5O and Figure S11). Most notably, G139R introduces a large and positively charged residue at the interface of two closely packed loops of the protomers of the homodimer, causing a steric clash and potentially disruption of the dimerization interface (Figure 5P). G176 is in a tight turn held by a salt bridge between the two neighboring residues T175 and R177, but G176 is also in close packing with the other protomer, and thus it is likely that the valine mutation in G176V causes a major disruption of both the protomer structure and the dimer (Figure 5Q). The E252K mutation is unlikely to be important for the integrity of the structure as the side chain points to the water phase (Figure 5R). We considered the possibility that in addition to preventing mitochondrial localization, G139R and G176V could also disrupt the formation of the dimer. To evaluate this, we attempted to perform oligomeric state analysis by SEC-MALLS, but this analysis proved to be impossible for the full-length proteins as the purification yields were low. Since only the N-terminal regulatory domains of citrin are involved in dimerization, we introduced the G176V mutation in the fusion of the N-terminal and C-terminal domains, which is produced as a dimer in *L. lactis* [13]. The mutant fusion protein was successfully expressed and purified from *L. lactis* in high yields, but it was monomeric with a molecular weight of 46.2 KDa (Figure 5S). Therefore, G176V disrupts the dimerization of the N-terminal domain. Notably, mutants G139R and G176V had a substantially altered thermostability profile, indicative of severe folding issues (Figure S12). Consequently, only a part of the purified protein population may have been folded, accounting for the lower transport activity. These observations are also consistent with the low purification yields of those mutants.

## 4 Discussion

As more mechanistic details are being revealed about the SLC25 family of mitochondrial carriers, several questions remain about its most complex members SLC25A12 and SLC25A13. Why are these carriers dimeric? Is each protomer functionally independent? What is the role of their additional N-terminal and C-terminal domains in the transport mechanism? Is calcium binding to the N-terminal domain regulating transport activity? Here, we have provided important mechanistic insights for most of these questions and new insights into the molecular cause of citrin deficiency, caused by the dysfunction of SLC25A13.

A major and most unexpected finding of this study is that the N-terminal domain of citrin is not responsible for the regulation of its transport activity via calcium binding and release and it is not essential for the transport activity either. Rather, we find that pathogenic mutations of the N-terminal domain affect the mitochondrial localization of citrin critically. The transport activity of full-length citrin is not regulated by calcium when tested *in vitro* on purified protein reconstituted in liposomes in contrast to previously published work, showing activation of reconstituted aralar from *D. melanogaster* by calcium [39]. We have performed a series of control experiments to establish the right conditions to make this assessment and to explain the discrepancy. In the complete absence of cations, no transport is observed with either wild-type citrin or the carrier domain alone (Figure S8), indicating that these conditions lead to destabilization of the proteoliposomes. A response to added calcium (Figure S8) is an artifact reflecting its beneficial effect on the stabilization of liposomes rather than the protein itself, as this effect can also be observed with the carrier domain alone. It is well known that divalent cations provide important counter charges to the negatively charged headgroups of lipids. To maintain stable proteoliposomes without calcium, we introduced 5 mM EGTA and 5 mM magnesium, providing 1.5 mM free magnesium available for the stabilization of the lipids. Under these conditions, calcium-free citrin was not activated upon addition of calcium, even at mM concentrations (Figure 4). Furthermore, a quadruple mutant with an inactivated calcium-binding site had transport activity comparable to that of wild-type citrin, indicating that lack of calcium binding does not affect transport. Thus, there does not seem to be a regulatory effect of calcium on transport activity.

Low nanomolar range affinity for calcium had been previously proposed for both citrin and aralar [14]. Indeed, EF-hand 2 is pre-formed in both paralogs, consistent with a high affinity site for calcium, irrespective of the state of the N-terminal domain [13]. EF-hand 2 is also non-canonical, as it is not part of an EF-hand pair, usually associated with conformational changes. Conformational changes in the N-terminal domain of aralar have been observed in the absence of calcium, but they only occurred when the amphipathic helix of the C-terminal domain was absent [13]. Taken together, it is unlikely that calcium has a regulatory effect on transport activity of citrin under physiologically relevant conditions. Thus, citrin is expected to be permanently active, as in most cells, calcium ion levels are approximately 100 nM and, even under stimulation of a cell signaling cascade, will not exceed the low micromolar range. The removal of the calcium-binding site also does not affect the import of citrin into the mitochondrial inner membrane. Therefore, the calcium-binding site could simply be a remnant of an EF-hand mechanism that has been lost in evolution. We also show that, the carrier domain of citrin alone is partly active, supporting the idea that the N-terminal domain is not critical for the substrate transport mechanism either, although its contribution to transport activity is not understood. The only other member of the SLC25 family that has a calcium-bound N-terminal domain is the mitochondrial ATP-Mg/Pi carrier [40,41]. In contrast to citrin, the activity of this carrier is calcium-regulated, which uses a very different mechanism. The N-terminal domain of the ATP-Mg/Pi carrier has two EF-hand pairs that release an amphipathic helix at low calcium levels, which subsequently binds to the carrier domain, inhibiting its activity [[42], [43], [44]].

**Figure 6:**
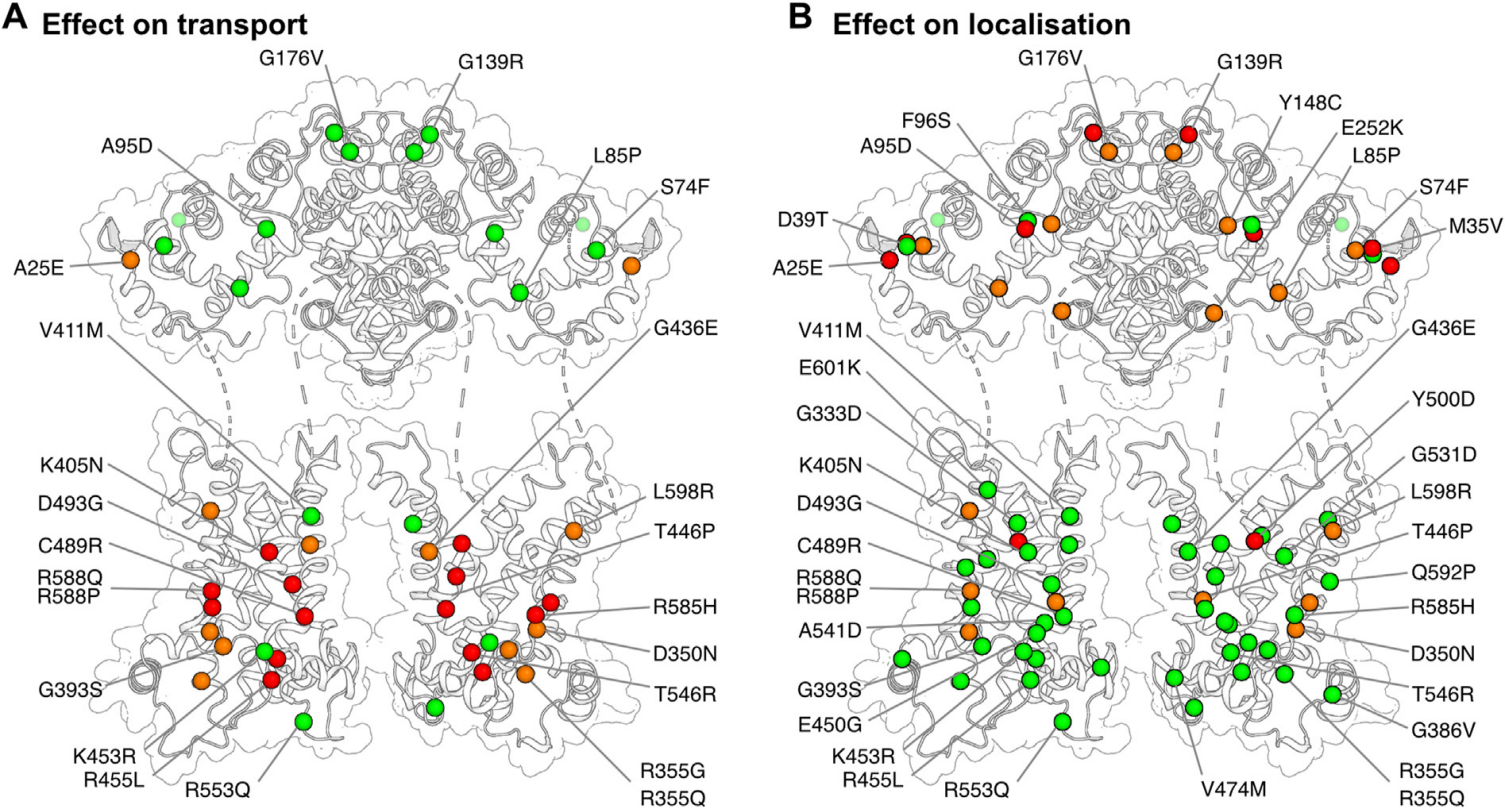
**Distinct pathogenic mechanisms for citrin variants.** (**A**) Effect of citrin pathogenic variants on transport. In the carrier domain, mutations, shown as spheres, affect several residues important for function, structural stability and conformational flexibility. Residues with more than 50% activity are shown in green spheres, with 5–50% activity with orange and with less that 5% activity in red. (**B**) Effect of citrin pathogenic variants on mitochondrial localization. Pathogenic mutations in the N-terminal domain primarily affect localization of citrin. Hierarchical clustering of cell type distribution has been used to categorize the mutations, shown as spheres. Green spheres represent variants with primarily mitochondrial distribution, with orange are variants with high levels of non-mitochondrial distribution and with red with primarily non-mitochondrial distribution.

By studying several pathogenic mutations of citrin, we show that mutations of the N-terminal domain cause mitochondrial localization defects. Most missense mutations in the N-terminal domain, namely A25E, M35V, S74F, L85P, A95D, Y148C, G139R, G176V, E252K, failed to localize to mitochondria efficiently. In contrast, most pathogenic mutations of the carrier domain had normal localization profiles (Figure 6). This observation suggests that pathogenicity of the disease variants of the N-terminal domain is driven primarily by defective localization. This observation was unexpected as the mitochondrial targeting signals for the SLC25 carrier proteins are likely to be in the carrier domains [45]. It is unclear how the mutations in the N-terminus impair protein import and localization and thus further studies are necessary to address some possible causes, such as mRNA stability, ribosome stalling, or protein targeting, folding or insertion issues.

Another outstanding question is why citrin and aralar are structural dimers, when all other mitochondrial carriers are likely to be structural monomers [46,47]. Interestingly, G176V, an N-terminal domain mutation located in the dimer interface, not only leads to a major mitochondrial localization defect but also disrupts the dimerization interface mediated by the N-terminal domains [13]. Another mutation, G136R, is also expected to disrupt the dimer interface, based on structural analysis. Since both pathogenic variants are still active for substrate transport, albeit with much lower transport rates, we hypothesize that the aspartate/glutamate carrier is a structural dimer rather than a functional dimer, where the two carrier domains function independently of each other. This notion is also supported by the observation that the carrier domain alone retains transport activity, despite the truncations of the N- and C-terminal domains. Interestingly, mutations that disrupt the dimer interface also led to extreme defects in mitochondrial localization. The connection between the two processes is yet to be understood.

Activity defects have been reported for pathogenic variants in other SLC25 members, which only have a carrier domain [7,[48], [49], [50]]. This functional impairment is usually either due to the loss of substrate coordination in the binding pocket or due to the inability of the protein to undergo the necessary conformational changes for substrate transport. In citrin, most of the pathogenic mutations in the carrier domain, had little or no effect on mitochondrial localization, but they had dramatic effects on transport activity (Figure 6). Some mutations did not impact function severely, for example, K453R and R553Q, but they are conservative replacements. Additionally, V411M and R553Q do not appear to impact the structure, mechanism, or localization of the protein, and thus they might be natural variants rather than pathogenic variants (Figure S13).

We note that the pathogenic mutations in the carrier and N-terminal domains of citrin generate distinct mechanisms of dysfunction in citrin deficiency. The first mechanism is caused by mutations in the carrier domain that dramatically impact transport activity, whereas the second is caused by mutations in the N-terminal domain that primarily disrupt mitochondrial localization (Figure 6). Beyond the missense mutations addressed in this study, citrin variants also include splicing site mutations, deletions, insertions, and nonsense mutations [3]. The consequences of these variants cannot currently be assessed, and their expression may be restricted by transcriptome control mechanisms, such as nonsense-mediated mRNA decay [51,52]. Studies in a small number of patients support the idea that the citrin protein is not expressed when mutations prevent full-length transcripts [53], but this has not been assessed for most cases. However, the genes of pathogenic variants involving missense mutations produce full-length transcripts and express citrin protein with a single amino acid substitution, and thus are expected to be present in citrin deficiency patients. Indeed, two variants have been also studied in patient fibroblasts and it was shown that they express at similar levels to wild-type citrin [54,55]. Along with providing novel insights into the mechanism of mitochondrial aspartate/glutamate carriers, this work may also facilitate diagnostic and prognostic assessments of citrin deficient patients.

## CRediT authorship contribution statement

**Sotiria Tavoulari:** Writing – review & editing, Writing – original draft, Visualization, Validation, Supervision, Methodology, Investigation, Formal analysis, Data curation, Conceptualization. **Denis Lacabanne:** Writing – review & editing, Writing – original draft, Visualization, Validation, Methodology, Investigation, Formal analysis, Data curation, Conceptualization. **Gonçalo C. Pereira:** Writing – review & editing, Visualization, Validation, Methodology, Investigation, Formal analysis, Data curation, Conceptualization. **Chancievan Thangaratnarajah:** Writing – review & editing, Visualization, Methodology, Investigation, Formal analysis, Data curation. **Martin S. King:** Writing – review & editing, Validation, Investigation, Formal analysis, Data curation. **Jiuya He:** Writing – review & editing, Methodology, Investigation, Formal analysis, Data curation. **Suvagata R. Chowdhury:** Writing – review & editing, Software, Methodology, Investigation, Formal analysis. **Lisa Tilokani:** Writing – review & editing, Methodology, Investigation, Formal analysis, Data curation. **Shane M. Palmer:** Writing – review & editing, Methodology, Investigation, Formal analysis, Data curation. **Julien Prudent:** Writing – review & editing, Validation, Methodology, Funding acquisition, Formal analysis, Conceptualization. **John E. Walker:** Writing – review & editing, Supervision, Funding acquisition, Formal analysis, Conceptualization. **Edmund R.S. Kunji:** Writing – review & editing, Writing – original draft, Visualization, Validation, Supervision, Project administration, Investigation, Funding acquisition, Formal analysis, Data curation, Conceptualization.

## Declaration of competing interest

The authors declare that they have no conflict of interest.

## Data Availability

Data will be made available on request.
