# Supplementary material for: Distinct roles for the domains of the mitochondrial aspartate/glutamate carrier citrin in organellar localization and substrate transport
Source: Mol Metab. 2024 Oct 16;90:102047. doi: 10.1016/j.molmet.2024.102047 (PMC11539162; doi:10.1016/j.molmet.2024.102047)
Supplement: Multimedia component 1 [file mmc1.docx]

**SUPPLEMENTARY INFORMATION**

**Distinct roles for the domains of the mitochondrial aspartate/glutamate carrier citrin in organellar localization and substrate transport**

**Authors:** Sotiria Tavoulari^1§*^, Denis Lacabanne^1§^, Gonçalo C. Pereira^1§^, Chancievan Thangaratnarajah^1^, Martin S. King^1^, Jiuya He^1^, Roy Chowdhury^1^, Lisa Tilokani^1^, Shane M. Palmer^1^, Julien Prudent^1^, John E. Walker^1^ and Edmund R.S. Kunji^1*^

**Institutions:**

^1^Medical Research Council Mitochondrial Biology Unit, University of Cambridge, Keith Peters Building, Cambridge Biomedical Campus, Hills Road, Cambridge, CB2 0XY United Kingdom.

**
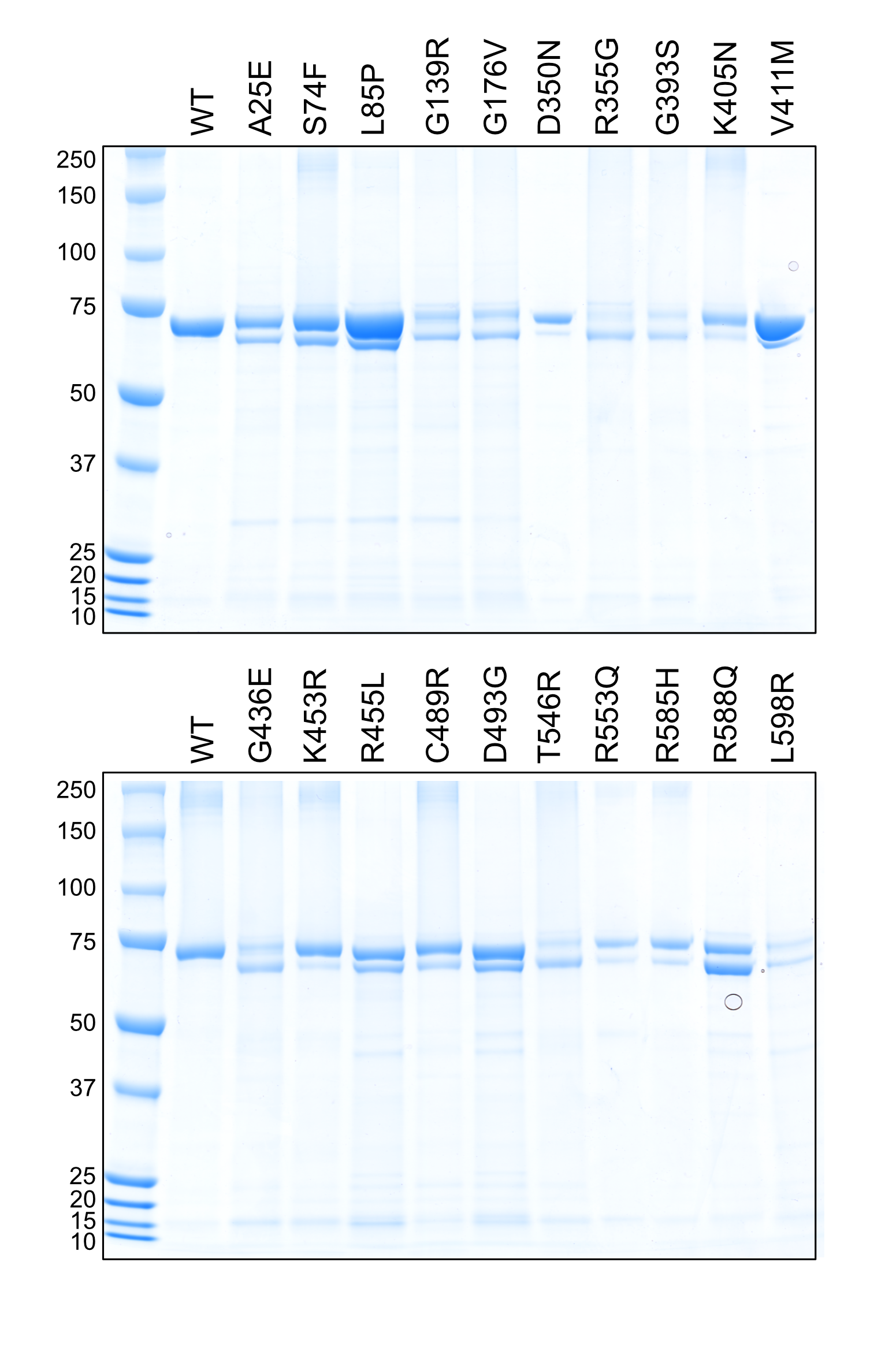
SUPPLEMENTARY FIGURES**

**Figure S1:** Purification of wild type citrin and variants. Each nickel affinity purified protein was analyzed by SDS–PAGE on 4-12% polyacrylamide gels and the bands were visualized by Coomassie Blue stain. Molecular weight (MW) markers are indicated. From the two major bands appearing on the gel in some mutants, the lower molecular weight band corresponds to Hsp60 as defined by mass spectrometry and has not been included on protein quantification in liposomes.

**
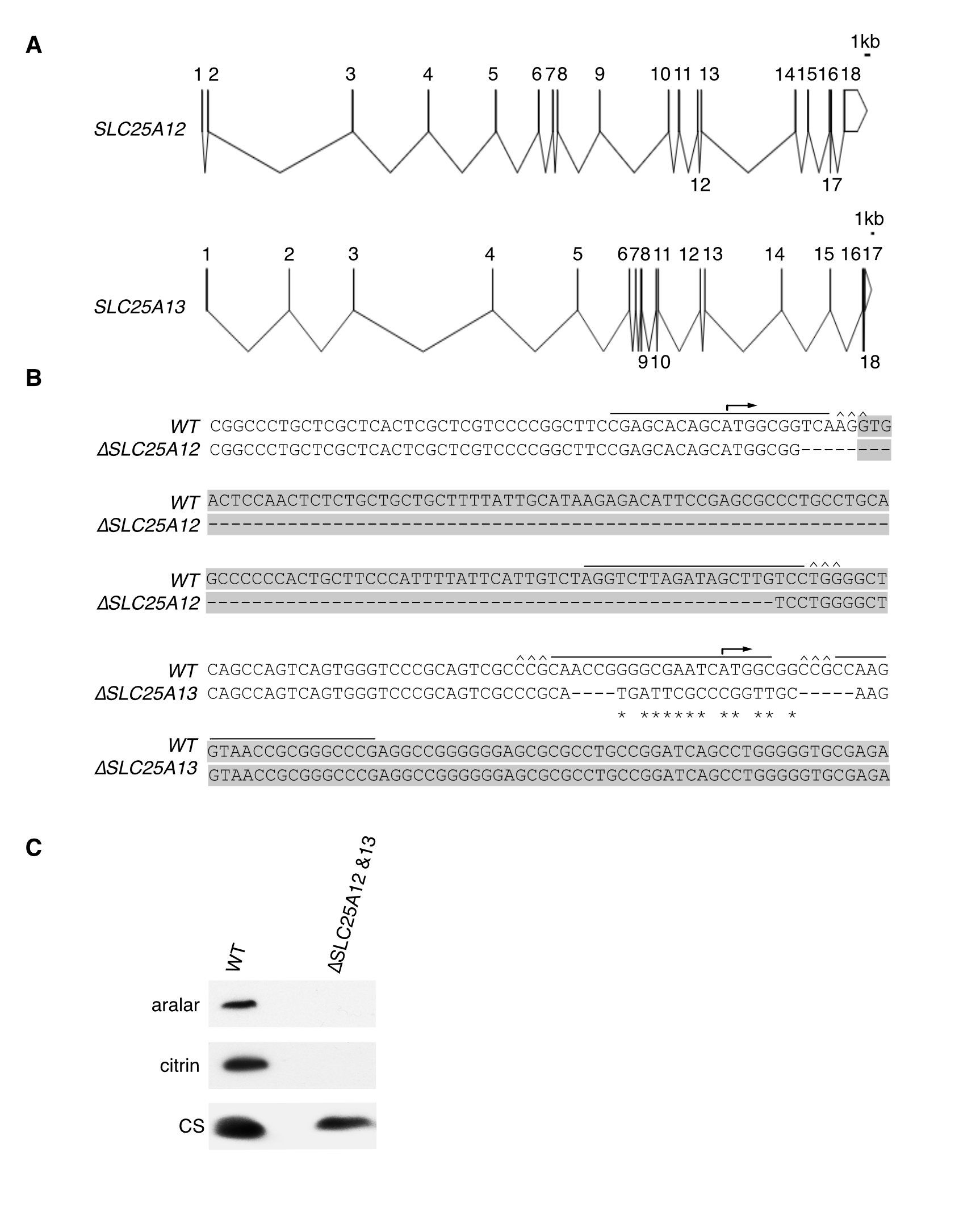
**

Figure S2: Design and validation of an aralar and citrin HAP1 knock-out cell line. (A) Structures of human genes SLC25A12 and SLC25A13 encoding aralar and citrin proteins respectively. Arabic numerals denote exons; black and unfilled areas in exons represent, respectively, protein coding and non-coding regions; intervening continuous lines between exons represent introns. On the right, the scale bars represent 1 kb. The exon-intron information was obtained from <http://www.ensembl.org>. The structures of the genes correspond to transcript ID references ENST00000422440.7 and ENST00000265631.10. Images were drawn with the Exon-Intron graphic maker (<http://wormweb.org/exonintron>). (B) The DNA sequences in the double knock-out ∆SLC25A12&13 are compared to the corresponding wild-type sequences for SLC25A12 and SLC25A13. Carets indicate the PAM (protospacer adjacent motif) sequences for each guide RNA, and solid lines the target sequences for guide RNAs. For gene editing, exon I was targeted in both genes. The targeted exon and sections of wild-type intron sequence (grey box) are aligned with the corresponding edited sequence. The horizontal arrows indicate the translational initiator methionine codons. Deleted DNA regions are denoted by dashed lines, and asterisks indicate non-matched bases which probably were random integrated during DNA repair. (C) Immunoblots of DDM extracts of mitoplasts from HAP1-wild-type and HAP1-ΔSLC25A12&13 cells, with antibodies against aralar and citrin on the left. Citrate synthase (CS) was used as a loading control.

**
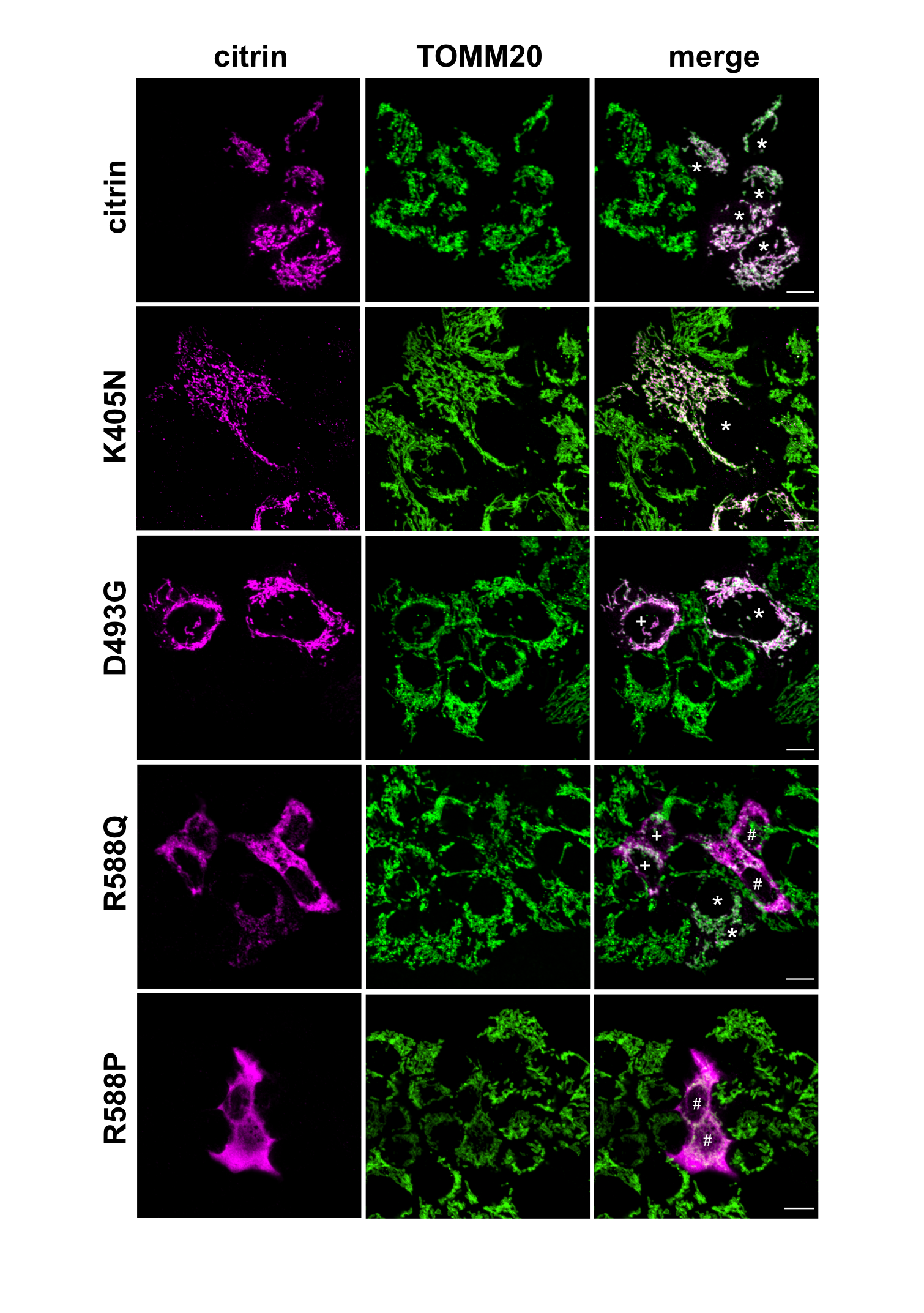
**

**Figure S3:** Representative confocal images of HAP1 citrin and aralar DKO expressing transiently wild type citrin or mutants in the predicted substrate binding site. For comparison, the signal intensity has been adjusted to that observed in cells expressing wild-type citrin. Total number of cells analyzed is shown in **Table S1**. Left column: citrin immunostaining, Middle column: TOMM20 immunostaining of mitochondria, Right column: merge. Type I cells are indicated by an asterisk (*), type II cells by a cross (+) and type III cells by a hashtag (#). Scale bars: 10 μm.

**
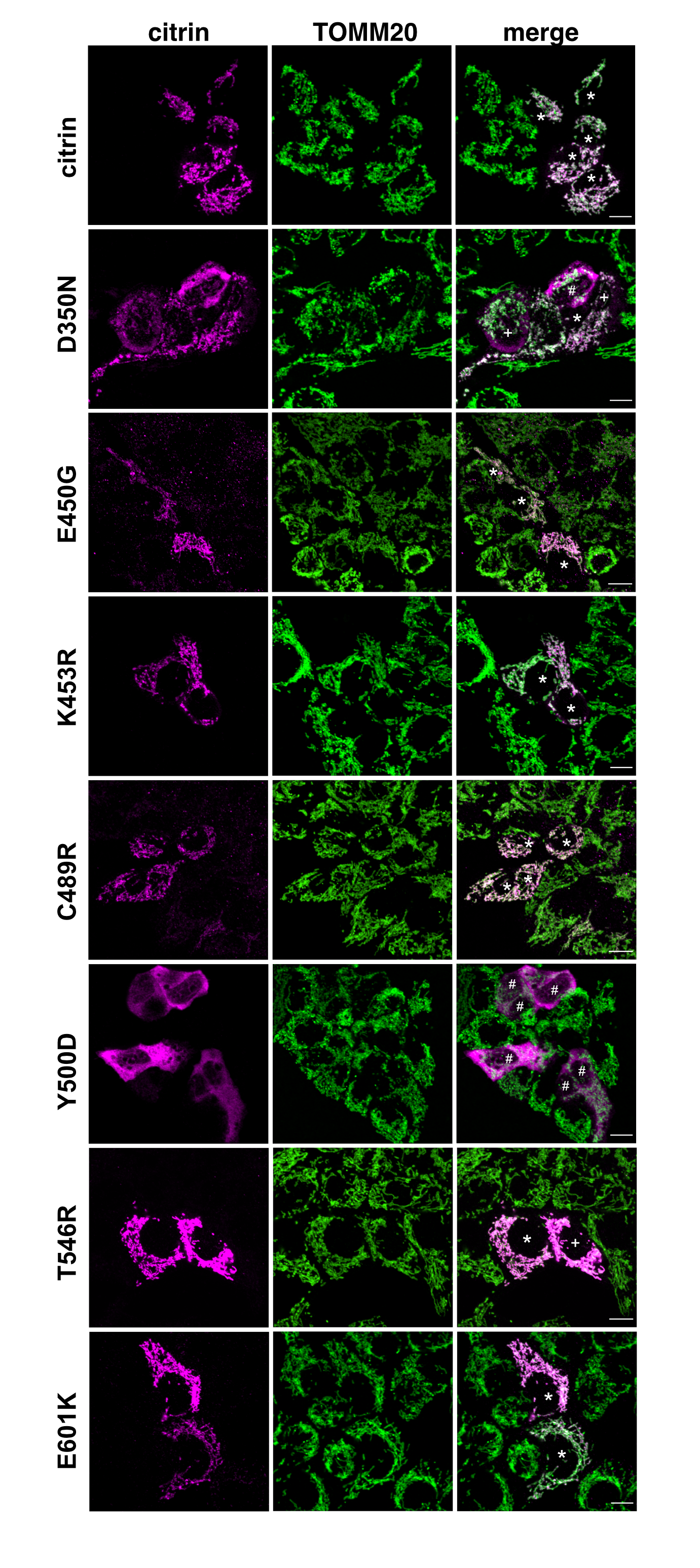
**

**Figure S4:** Representative confocal images of HAP1 citrin and aralar DKO expressing wild-type citrin or mutants in the matrix and cytoplasmic networks. Image analysis was performed as in **Figure S3**. Total number of cells analyzed is shown in **Table S1**. Left column: citrin immunostaining, Middle column: TOMM20 immunostaining of mitochondria, Right column: merge. Type I cells are indicated by an asterisk (*), type II cells by a cross (+) and type III cells by a hashtag (#). Scale bars: 10 μm.

**
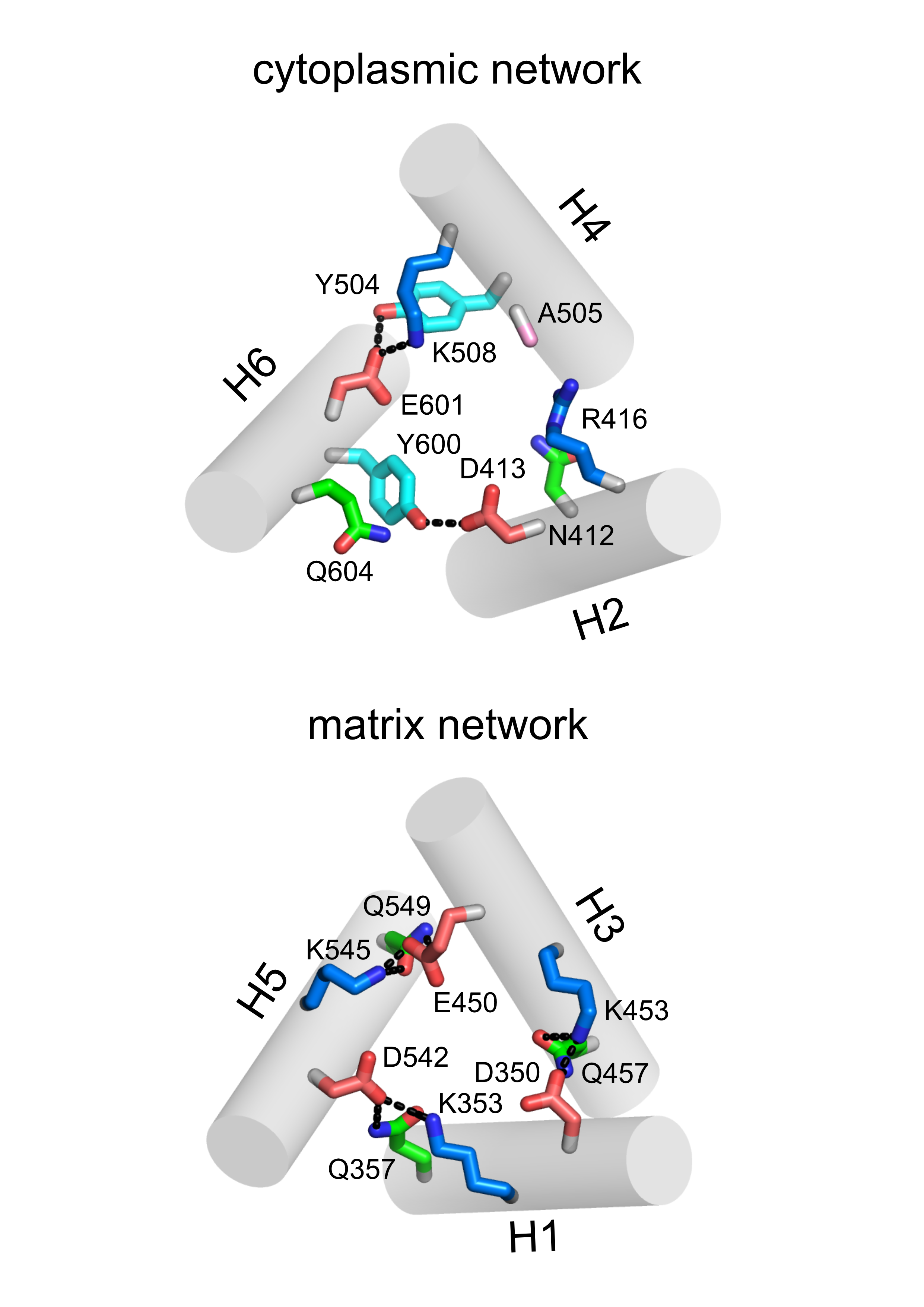
**

**Figure S5**: Cytoplasmic (top) and matrix (bottom) salt bridge network of the aspartate/glutamate carrier. Positively charged, negatively charged, polar, aliphatic, and aromatic residues are shown in blue, red, green, pink, and cyan colors, respectively. The bonds are shown as black dashes. The model of the cytoplasmic network is derived from PDB entry 6gci (chain A), and the matrix network from PDB entry 1okc.

**
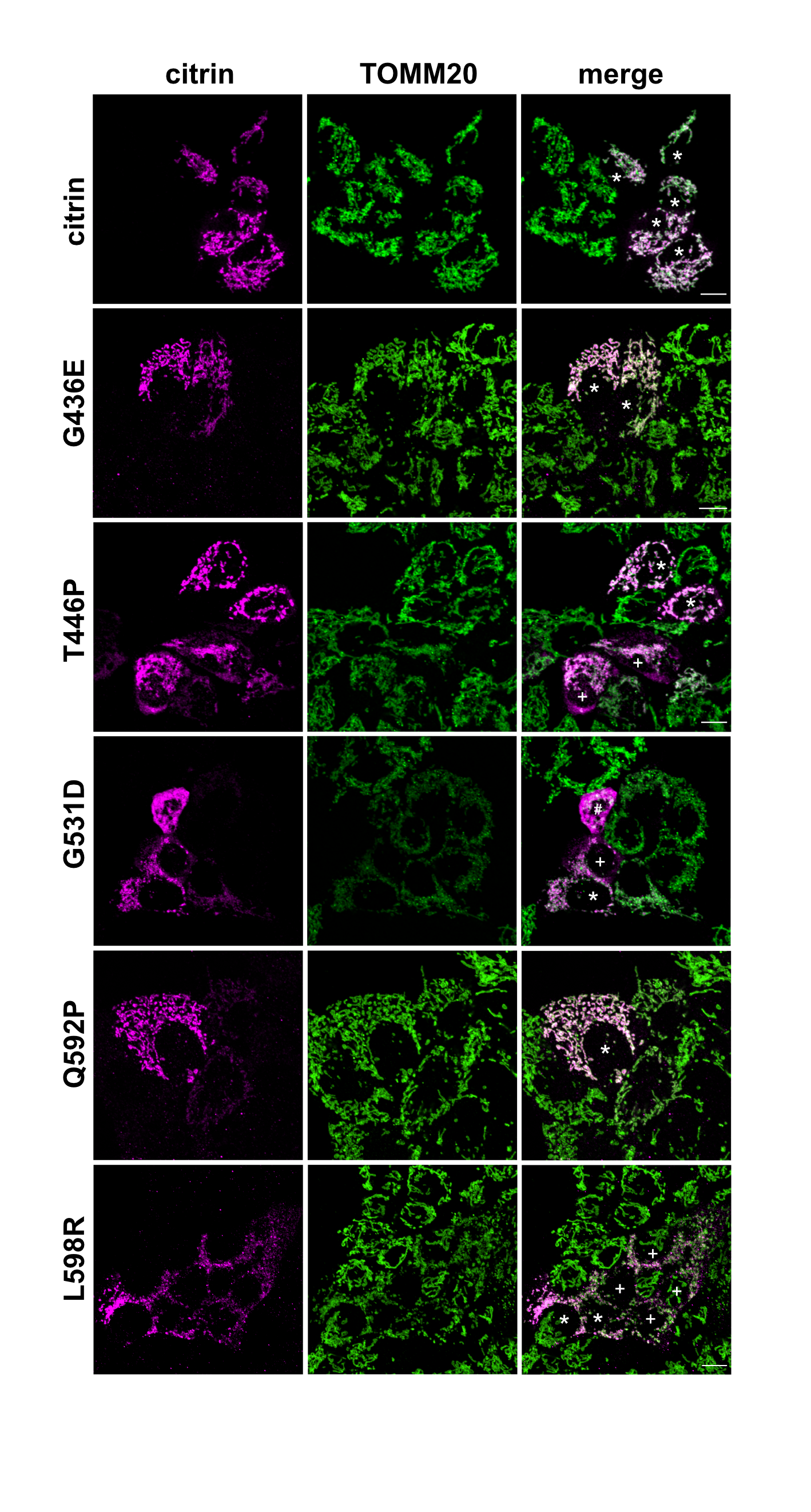
Figure S6:** Representative confocal images of HAP1 citrin and aralar DKO expressing wild-type citrin or mutants important for protein dynamics. Image analysis was performed as in **Figure S3**. Total number of cells analyzed is shown in **Table S1**. Left column: citrin immunostaining, Middle column: TOMM20 immunostaining of mitochondria, Right column: merge. Type I cells are indicated by an asterisk (*), type II cells by a cross (+) and type III cells by a hashtag (#). Scale bars: 10 μm.

**
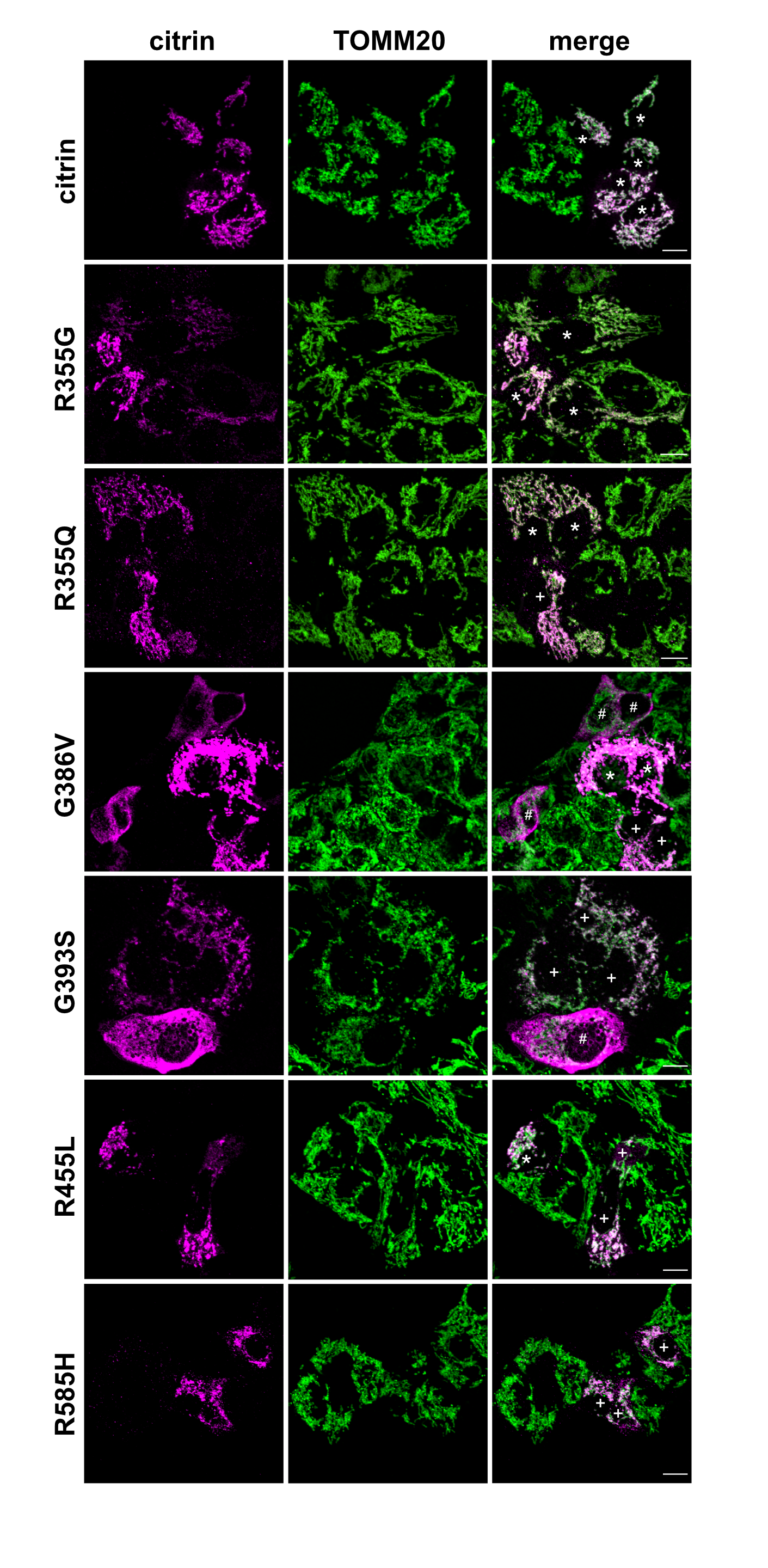
Figure S7:** Representative confocal images of HAP1 citrin and aralar DKO expressing wild-type citrin or mutants in positions important for structural integrity. Image analysis was performed as in **Figure S3**. Total number of cells analyzed is shown in **Table S1**. Left column: citrin immunostaining, Middle column: TOMM20 immunostaining of mitochondria, Right column: merge. Type I cells are indicated by an asterisk (*), type II cells by a cross (+) and type III cells by a hashtag (#). Scale bars: 10 μm.

**Figure S8:** Calcium addition in liposomes prepared free of divalent cations leads to protein-independent proteoliposome activation. (**A**) Citrin proteoliposomes, prepared in the absence of cations, respond to addition of increasing calcium concentrations in an aspartate homo-exchange assay. (**B**) Citrin carrier domain, prepared in the absence of cations, respond to addition of increasing calcium concentrations in an aspartate homo-exchange assay.

**
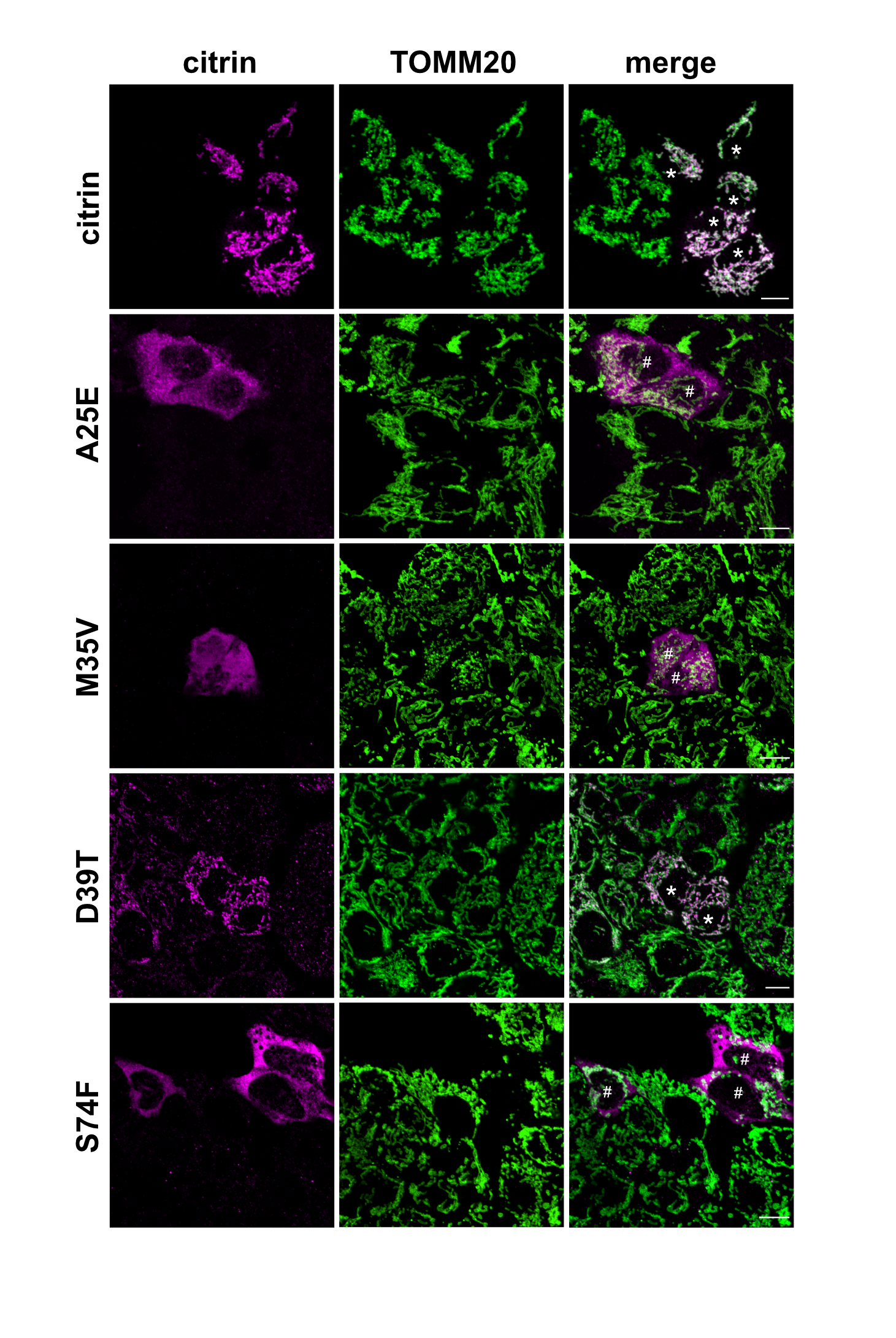
Figure S9:** Representative confocal images of HAP1 citrin and aralar DKO expressing wild-type citrin or mutants in the mobile unit of the N-terminal domain. Image analysis was performed as in **Figure S3**. Total number of cells analyzed is shown in **Table S1**. Left column: citrin immunostaining, Middle column: TOMM20 immunostaining of mitochondria, Right column: merge. Type I cells are indicated by an asterisk (*), type II cells by a cross (+) and type III cells by a hashtag (#). Scale bars: 10 μm.

**
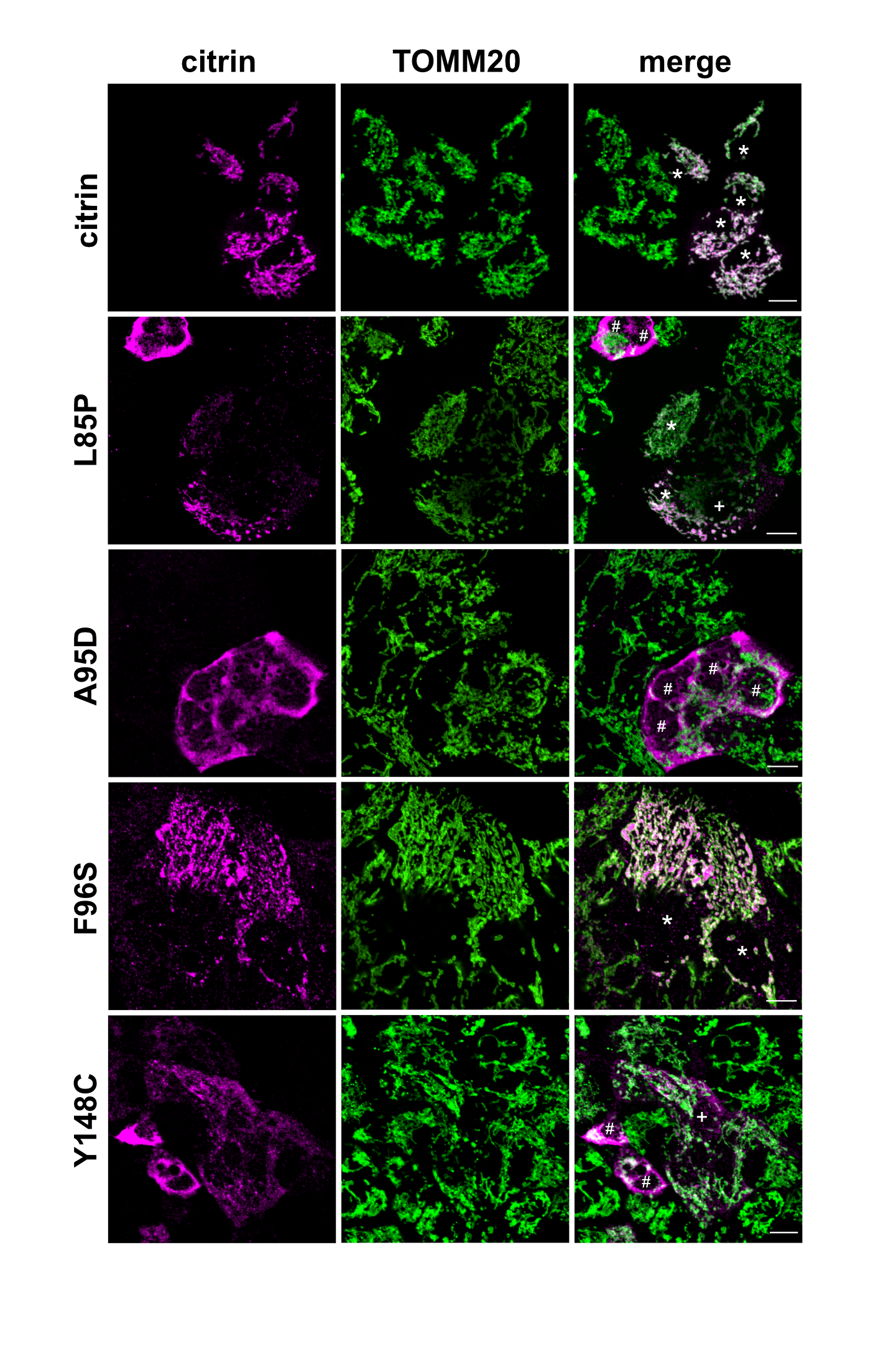
Figure S10:** Representative confocal images of HAP1 citrin and aralar DKO expressing wild-type citrin or mutants in the hinge region of the N-terminal domain. Image analysis was performed as in **Figure S3**. Total number of cells analyzed is shown in **Table S1**. Left column: citrin immunostaining, Middle column: TOMM20 immunostaining of mitochondria, Right column: merge. Type I cells are indicated by an asterisk (*), type II cells by a cross (+) and type III cells by a hashtag (#). Scale bars: 10 μm.

**
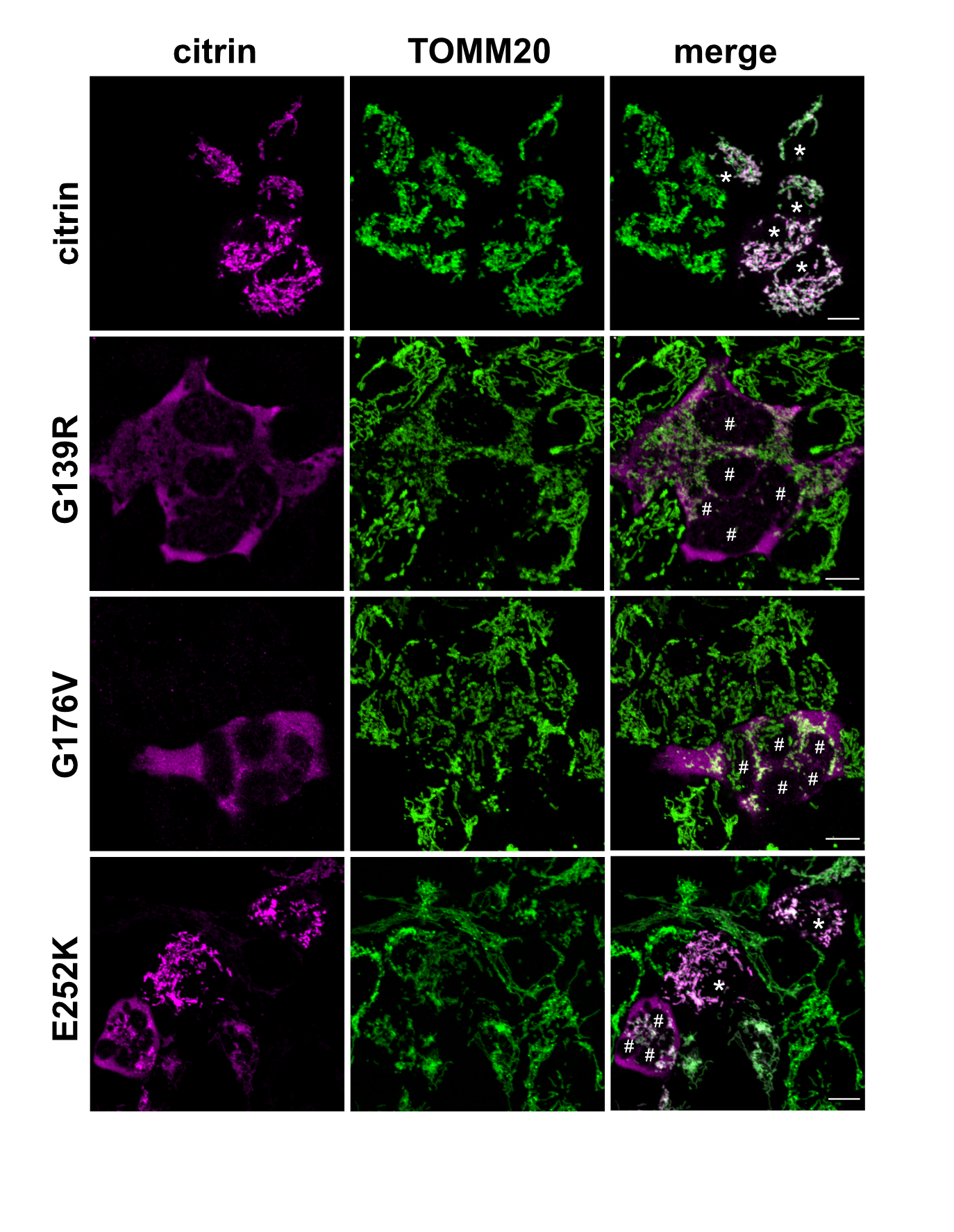
Figure S11:** Representative confocal images of HAP1 citrin and aralar DKO expressing wild-type citrin or mutants in the dimer interface of the N-terminal domain. Image analysis was performed as in **Figure S3**. Total number of cells analyzed is shown in **Table S1.** Left column: citrin immunostaining, Middle column: TOMM20 immunostaining of mitochondria, Right column: merge. Type I cells are indicated by an asterisk (*), type II cells by a cross (+) and type III cells by a hashtag (#). Scale bars: 10 μm.

**
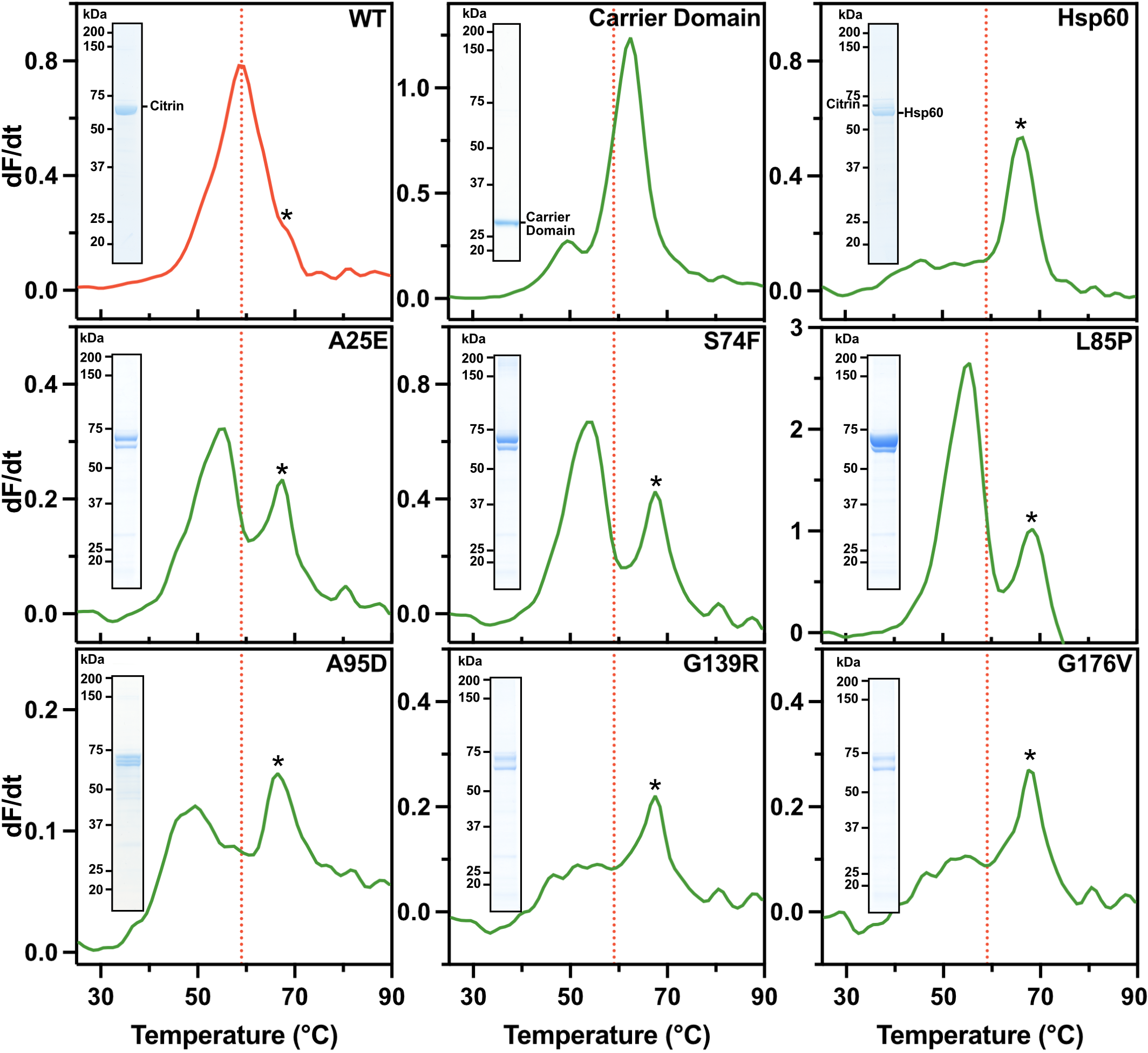
**

**Figure S12.** Thermostability analysis by CPM for wild-type citrin, the carrier domain alone and variants of the N-terminal domain. The thermostability profile of Hsp60, which usually co-purifies with citrin (Figure S1), is also shown. Dotted lines indicate the apparent melting temperature for wild-type citrin. Asterisks indicate the co-purified Hsp60. The results shown are from a representative experiment, performed twice. The inserts show SDS-PAGE analysis of the purified protein stained by Coomassie Blue, showing also the molecular weight (MW) markers.

**
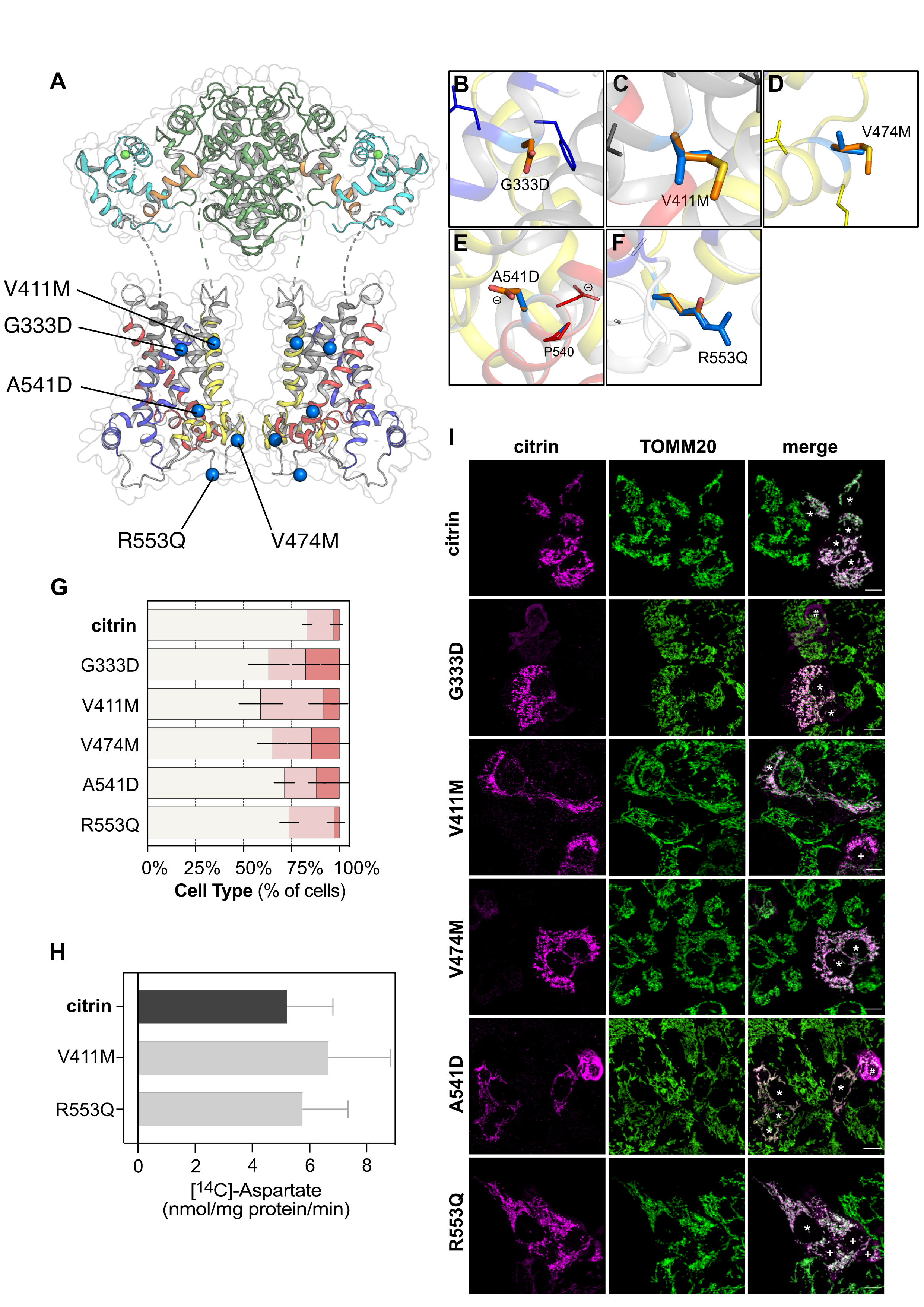
**

**Figure S13.** (**A**) Structural model of citrin. Marine spheres indicate the positions of residues mutated in the protein surface. (**B-F**) Citrin mutations, shown in a structural context. The wild-type residue is shown in marine stick representation and the mutation in orange. (**G**) Distribution of cytosolic and mitochondrial citrin and disease variants, classified into three types (n=7 for citrin wild type, n=3-4 for variants), as described in **Figure 2.** Color coding is as in **Figure 2**. Error bars represent the standard error of the mean. (**H**) Time course of aspartate homo-exchange in proteoliposomes. Initial rates of transport for wild type were compared with those of V411M and R553Q (n=12 for citrin wild type, n=3 for variants). Error bars represent the standard deviation. (**I**) Representative confocal images of HAP1 citrin and aralar DKO expressing wild-type citrin or mutations at the surface residues. Image analysis was performed as in **Figure S3**. Total number of cells analyzed is shown in **Table S1**. Left column: citrin immunostaining, Middle column: TOMM20 immunostaining of mitochondria, Right column: merge. Type I cells are indicated by an asterisk (*), type II cells by a cross (+) and type III cells by a hashtag (#). Scale bars: 10 μm.

**Supplementary Tables**

**Table S1. Number of cells analyzed to determine the distribution of mitochondrial and cytosolic citrin in type I, II and III cells.**

|  | **Total number of cells analyzed** |
| --- | --- |
| **Citrin WT** | 180 |
| **A25E** | 49 |
| **M35V** | 46 |
| **D39T** | 30 |
| **S74F** | 56 |
| **L85P** | 81 |
| **A95D** | 48 |
| **F96S** | 59 |
| **G139R** | 89 |
| **Y148C** | 103 |
| **G176V** | 71 |
| **E252K** | 119 |
| **G333D** | 84 |
| **D350N** | 84 |
| **R355G** | 89 |
| **R355Q** | 68 |
| **G386V** | 52 |
| **G393S** | 68 |
| **K405N** | 69 |
| **V411M** | 69 |
| **G436E** | 56 |
| **T446P** | 81 |
| **E450G** | 52 |
| **K453R** | 69 |
| **R455L** | 81 |
| **V474M** | 68 |
| **C489R** | 86 |
| **D493G** | 52 |
| **Y500D** | 64 |
| **G531D** | 87 |
| **A541D** | 80 |
| **T546R** | 64 |
| **R553Q** | 57 |
| **R585H** | 85 |
| **R588P** | 58 |
| **Q592Q** | 105 |
| **L598R** | 77 |
| **E601K** | 54 |

**Table S2. Expression levels of mutant variants in HAP1 cells.**

Cellular citrin signal was normalized to the corresponding TOMM20 signal and differences between mutant variants and wild type citrin were calculated by a two-way ANOVA, as described in Material and Methods. Data shown is for Class I only cells or for all cells analyzed (Classes I to III). Significance was accepted at 5% and p-values for statistically significant differences are indicated in bold. N represents the number of biological repeats.

|  | Class I only | | | Class I-III | | |  |  |
| --- | --- | --- | --- | --- | --- | --- | --- | --- |
| Variant | Mean | SEM | N | Mean | SEM | N | p-value vs citrin | Total number of cells |
| **Citrin WT** | 144.5 | 19.7 | 7 | 164.2 | 22.3 | 7 |  | 170 |
| **A25E** | 35.6 | 0.0 | 1 | 107.2 | 40.2 | 3 | **0.0377** | 52 |
| **M35V** | 59.8 | 0.0 | 1 | 104.3 | 22.4 | 3 | 0.0565 | 56 |
| **D39T** | 66.2 | 5.61 | 2 | 65.9 | 5.35 | 2 | **0.0055** | 45 |
| **S74F** | 72.5 | 29.2 | 2 | 124.1 | 1.68 | 3 | 0.0766 | 65 |
| **L85P** | 108.5 | 18.5 | 4 | 139.5 | 26.2 | 4 | 0.2196 | 91 |
| **A95D** | 120.9 | 25.2 | 2 | 144.0 | 51.0 | 3 | 0.4876 | 50 |
| **F96S** | 135.5 | 46.3 | 2 | 172.0 | 48.9 | 2 | 0.9853 | 56 |
| **G139R** | 83.4 | 34.4 | 2 | 140.2 | 35.9 | 4 | 0.1855 | 88 |
| **Y148C** | 132.4 | 29.7 | 3 | 165.0 | 32.6 | 3 | 0.8349 | 102 |
| **G176V** | 53.5 | 11.0 | 4 | 85.2 | 9.25 | 4 | **0.0007** | 73 |
| **E252K** | 193.0 | 53.5 | 4 | 208.2 | 43.5 | 4 | 0.0615 | 130 |
| **G333D** | 147.9 | 38.0 | 4 | 152.2 | 39.5 | 4 | 0.8629 | 109 |
| **D350N** | 116.6 | 24.5 | 3 | 143.2 | 27.4 | 3 | 0.3689 | 82 |
| **R355G** | 170.0 | 36.8 | 3 | 174.0 | 44.2 | 3 | 0.5156 | 97 |
| **R355Q** | 117.7 | 18.0 | 3 | 154.9 | 32.1 | 3 | 0.5062 | 74 |
| **G386V** | 131.4 | 13.0 | 3 | 139.9 | 16.0 | 3 | 0.4916 | 50 |
| **G393S** | 123.9 | 19.0 | 3 | 150.4 | 15.3 | 3 | 0.5276 | 74 |
| **K405N** | 157.3 | 70.9 | 3 | 163.8 | 69.6 | 3 | 0.8189 | 74 |
| **V411M** | 111.9 | 17.9 | 3 | 138.6 | 13.2 | 3 | 0.2844 | 69 |
| **G436E** | 153.8 | 43.3 | 3 | 156.3 | 40.6 | 3 | 0.9797 | 57 |
| **T446P** | 158.4 | 20.1 | 3 | 198.7 | 32.1 | 3 | 0.3731 | 81 |
| **E450G** | 145.9 | 58.9 | 2 | 162.2 | 75.5 | 2 | 0.9918 | 56 |
| **K453R** | 162.3 | 33.9 | 3 | 173.2 | 35.6 | 3 | 0.6212 | 80 |
| **R455L** | 179.2 | 55.9 | 3 | 167.4 | 33.4 | 3 | 0.4844 | 76 |
| **V474M** | 152.7 | 15.8 | 3 | 146.0 | 9.25 | 3 | 0.8543 | 66 |
| **C489R** | 176.4 | 35.8 | 3 | 181.6 | 44.6 | 3 | 0.3637 | 109 |
| **D493G** | 156.3 | 62.4 | 3 | 173.8 | 62.3 | 3 | 0.6933 | 57 |
| **Y500D** | 93.6 | 28.1 | 3 | 136.0 | 24.7 | 3 | 0.1462 | 42 |
| **G531D** | 163.1 | 28.2 | 3 | 190.6 | 38.2 | 3 | 0.4073 | 94 |
| **A541D** | 134.2 | 11.9 | 2 | 149.9 | 11.5 | 3 | 0.6979 | 82 |
| **T546R** | 144.8 | 27.3 | 3 | 166.8 | 31.2 | 3 | 0.9579 | 67 |
| **R553Q** | 155.1 | 12.0 | 3 | 177.4 | 8.39 | 3 | 0.6601 | 62 |
| **R585H** | 160.0 | 1.88 | 3 | 170.7 | 9.82 | 3 | 0.6849 | 65 |
| **R588Q** | 166.1 | 39.1 | 3 | 177.1 | 36.4 | 3 | 0.5242 | 95 |
| **R588P** | 51.1 | 4.72 | 3 | 109.7 | 44.1 | 3 | **0.0198** | **61** |
| **Q592P** | 208.0 | 26.9 | 3 | 228.9 | 31.0 | 3 | **0.0189** | **59** |
| **L598R** | 100.3 | 20.2 | 4 | 120.6 | 15.6 | 4 | 0.0761 | 73 |
| **E601K** | 155.5 | 57.2 | 3 | 135.0 | 39.6 | 3 | 0.7381 | 58 |

**Table S3. Total and free calcium concentrations in samples analyzed for transport**

Total Calcium analysis was carried out using an 8900 Triple Quadrupole Inductively Coupled Plasma Mass Spectrometer and free-calcium concentration was then estimated using Maxchelator (https://somapp.ucdmc.ucdavis.edu/pharmacology/bers/maxchelator/).

|  | **Samples** | **Total Ca (µmol/L)** | **Free Ca (pmol/L)** |
| --- | --- | --- | --- |
| Repeat 1 | D66A/T68A/D70A/E77A | 6.28 | 58 |
| Repeat 1 | Citrin WT | 6.09 | 56 |
| Repeat 2 | D66A/T68A/D70A/E77A | 2.1 | 29 |
| Repeat 2 | Citrin WT | 3.12 | 29 |
| Repeat 3 | D66A/T68A/D70A/E77A | 2.43 | 22 |
| Repeat 3 | Citrin WT | 2.57 | 24 |
| Repeat 4 | D66A/T68A/D70A/E77A | 4.45 | 41 |
| Repeat 4 | Citrin WT | 4.07 | 38 |
| Repeat 4 | Citrin WT | 2.41 | 22 |
